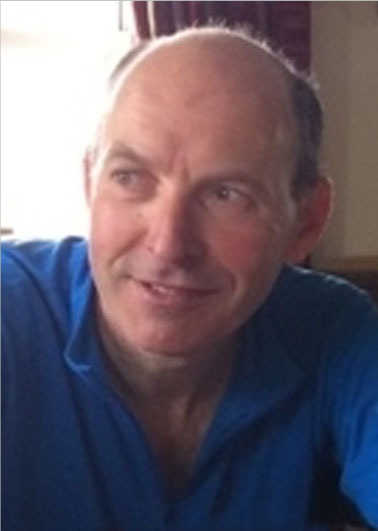# Iridium‐Catalysed C−H Borylation of Heteroarenes: Balancing Steric and Electronic Regiocontrol

**DOI:** 10.1002/anie.202001520

**Published:** 2020-11-03

**Authors:** Jay S. Wright, Peter J. H. Scott, Patrick G. Steel

**Affiliations:** ^1^ Department of Chemistry University of Durham Science Laboratories, South Road Durham Durham DH1 3LE UK; ^2^ Department of Radiology University of Michigan Ann Arbor Michigan USA

**Keywords:** borylation, catalysis, heteroarenes, iridium, regioselectivity

## Abstract

The iridium‐catalysed borylation of aromatic C−H bonds has become the preferred method for the synthesis of aromatic organoboron compounds. The reaction is highly efficient, tolerant of a broad range of substituents and can be applied to both carbocyclic and heterocyclic substrates. The regioselectivity of C−H activation is dominated by steric considerations and there have been considerable efforts to develop more selective processes for less constrained substrates. However, most of these have focused on benzenoid‐type substrates and in contrast, heteroarenes remain much desired but more challenging substrates with the position and/or nature of the heteroatom(s) significantly affecting reactivity and regioselectivity. This review will survey the borylation of heteroarenes, focusing on the influence of steric and electronic effects on regiochemical outcome and, by linking to current mechanistic understandings, will provide insights to what is currently possible and where further developments are required.

## Graphical abstract

**Which C−H bond?** The iridium‐catalysed C−H borylation reaction is a powerful method for the preparation of aromatic organoboronate esters. Sterically regulated regioselectivity dominates carbocyclic aromatic C−H borylation. In contrast, heterocyclic aromatics display a much greater influence from electronic effects. In this review, examples of heterocyclic C−H borylation are surveyed, and the origins of heterocyclic C−H borylation regioselectivities discussed.

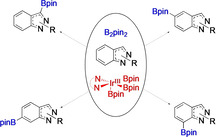

## 1 Introduction

Compounds bearing heteroaromatic scaffolds feature prevalently in pharmaceuticals, bioactive molecules, ligands for metal complexes, natural products, agrochemicals, and other functional materials.[^1^, ^2^, ^3^, ^4^, ^5^, ^6^, ^7^] Therefore, atom‐economical, streamlined syntheses of these molecules are of commercial value. Most heteroarenes are traditionally prepared by de novo synthesis and variation of substitution patterns can often require considerable synthetic effort. Consequently, methods that enable late‐stage modification have become desirable. In particular, C−H activation strategies that improve overall atom‐ and step‐economy have attracted the attention of many research groups in both academic and industrial settings, and numerous synthetic procedures for the formation of carbon–carbon and carbon–heteroatom bonds based on C−H bond activation strategies have been developed. Reflecting the versatility enabled by a C−B bond, iridium‐catalysed C−H borylation has become a major option for this chemistry. However, the regioselectivity of heteroarene C−H borylation can be challenging to predict and rationalise, and this review summarises current understanding of this important transformation.

## 2 Aromatic Boronate Esters

### 2.1 Introduction to Organoboron Compounds

**Figure 1:**
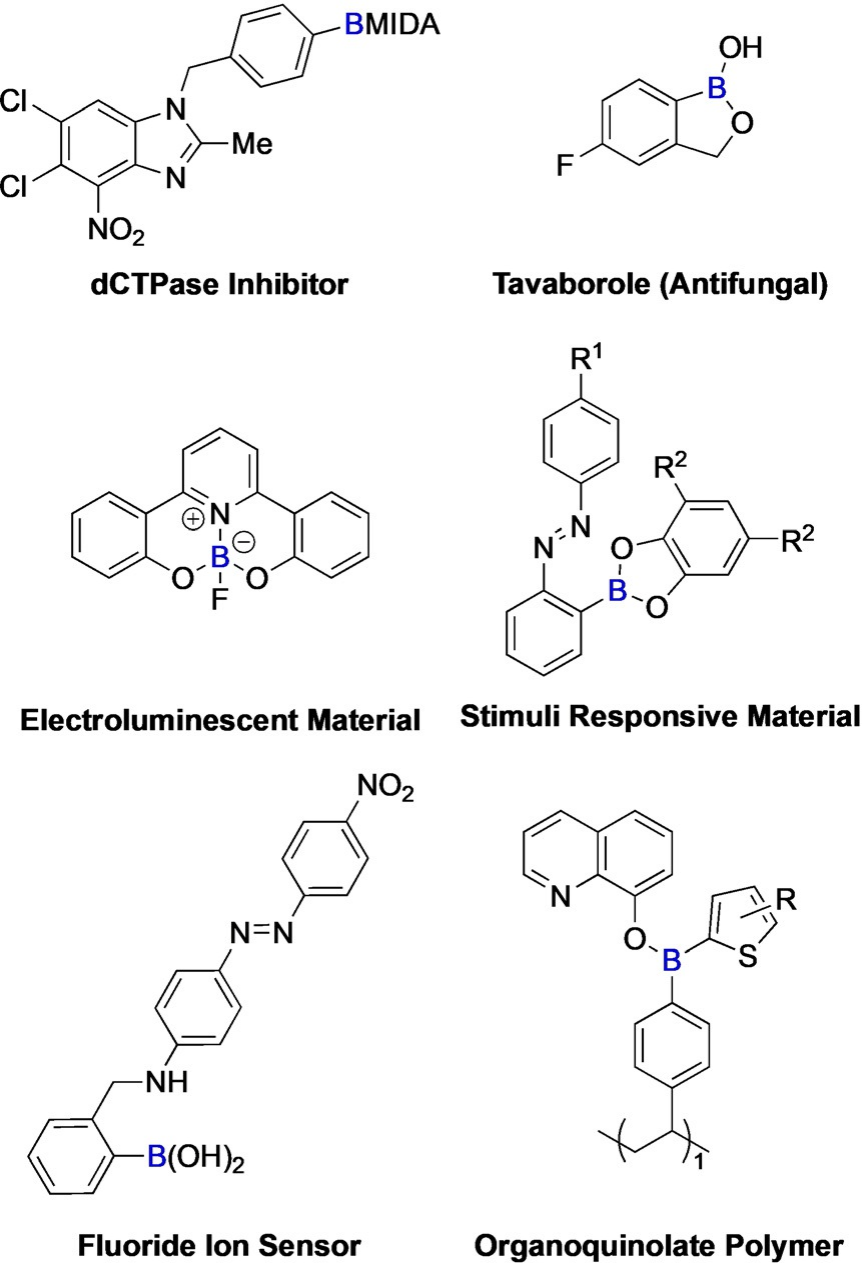
Applications of organoboron compounds. MIDA=*N*‐methyliminodiacetic acid.

**Figure 2:**
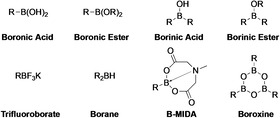
Selected classes of organoboron compounds.

Although organoboron compounds do not appear in nature, applications are emerging in radiochemistry, chemical biology, and medicinal chemistry as well as polymers and other functional materials (Figure 1).[^8^, ^9^, ^10^, ^11^, ^12^, ^13^, ^14^, ^15^, ^16^, ^17^, ^18^] However, the greatest use of these compounds resides in their use as reagents for synthesis. In 1979, Suzuki and Miyaura reported that organoboron compounds could be cross‐coupled with organohalides to form C−C bonds with catalytic quantities of Pd.[^19^, ^20^] This is now the second most practiced reaction in medicinal chemistry and natural product synthesis.^[21]^ Subsequently, many other useful transformations of the C−B bond have been developed,[^22^, ^23^, ^24^, ^25^, ^26^, ^27^] which has secured the status of organoboron compounds as important intermediates in synthesis. A variety of organoboron derivatives (Figure 2) including boranes, boronic acids, boronic (boronate) esters, borinic acids, borinic esters, boroxines, and trifluoroborates have been employed in these roles, with the boronate ester being the most frequently used. This reflects their ease of handling, good reactivity, and solubility and, when compared with alternative organometallic analogues, such as organostannane, organozinc and organocopper reagents, greater air stability, lower toxicity, and commercial availability.[^28^, ^29^] Whilst alkyl and alkenyl boronate compounds are widely used and find growing application, the most important class of boronate esters are the aromatic derivatives.

### 2.2 Synthesis of Aromatic Organoboron Compounds

**Scheme 1:**
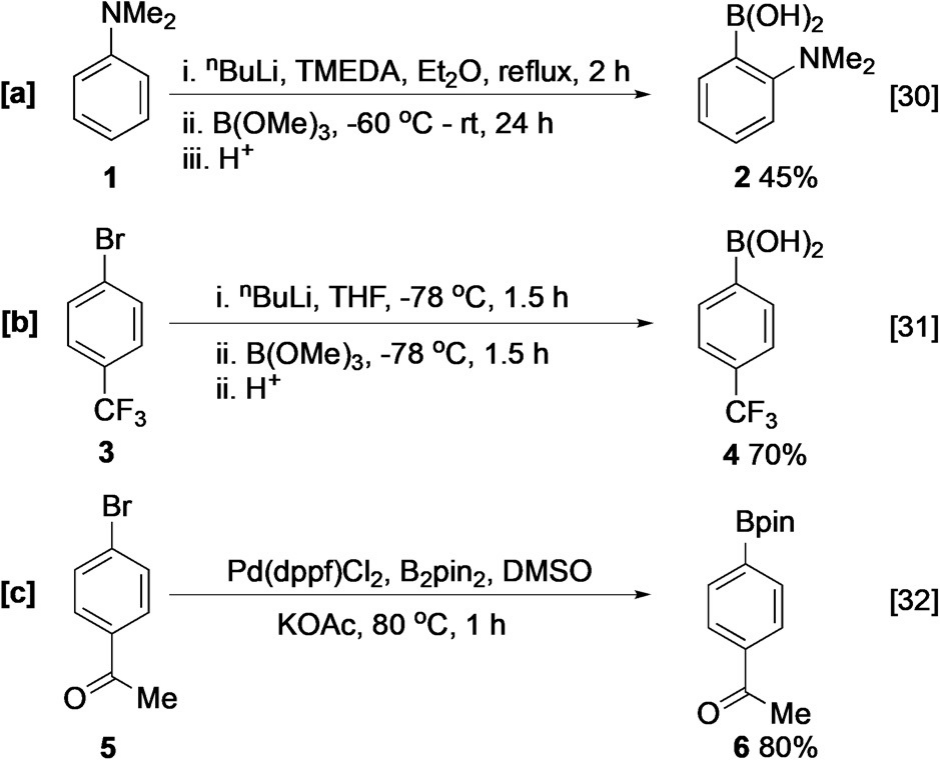
Selected syntheses of aryl organoboron compounds; literature references given in square brackets. dppf=1,1′‐bis(diphenylphosphino)ferrocene, pin=pinacolyl, TMEDA=tetramethylethylenediamine.

Traditionally, aromatic boronate esters have been synthesised by metalation of a C−H or C−X bond (X=Cl, Br, I) by a representative organometallic reagent, followed by reaction with a borate ester (Scheme 1 a,b).[^30^, ^31^] Whilst this strategy carries advantages, such as low reagent cost and operational simplicity, there are limitations. For instance, in C−H metalation a directing/activating group can be required to provide C−H reactivity and selectivity. This is less problematic in metal–halogen exchange, which is typically faster than C−H deprotonation. However, prefunctionalisation is required to generate the haloarene precursor. Furthermore, the hard bases required offer poor functional group tolerance. In this context, transition metal catalysts are attractive because they can offer superior scope, milder reaction conditions, and improved atom economy (Scheme 1 c).[^27^, ^32^, ^33^, ^34^] Whilst this approach is amenable to late‐stage functionalisation, it remains limited by the requirement for a prefunctionalised aromatic halide.

A simpler approach to borylation involves the direct transformation of a C−H to a C−B bond. To a significant extent, catalytic borylation of aromatic C−H bonds has addressed many of the shortcomings of these other strategies.

## 3 Aromatic C−H Borylation

**Scheme 2:**
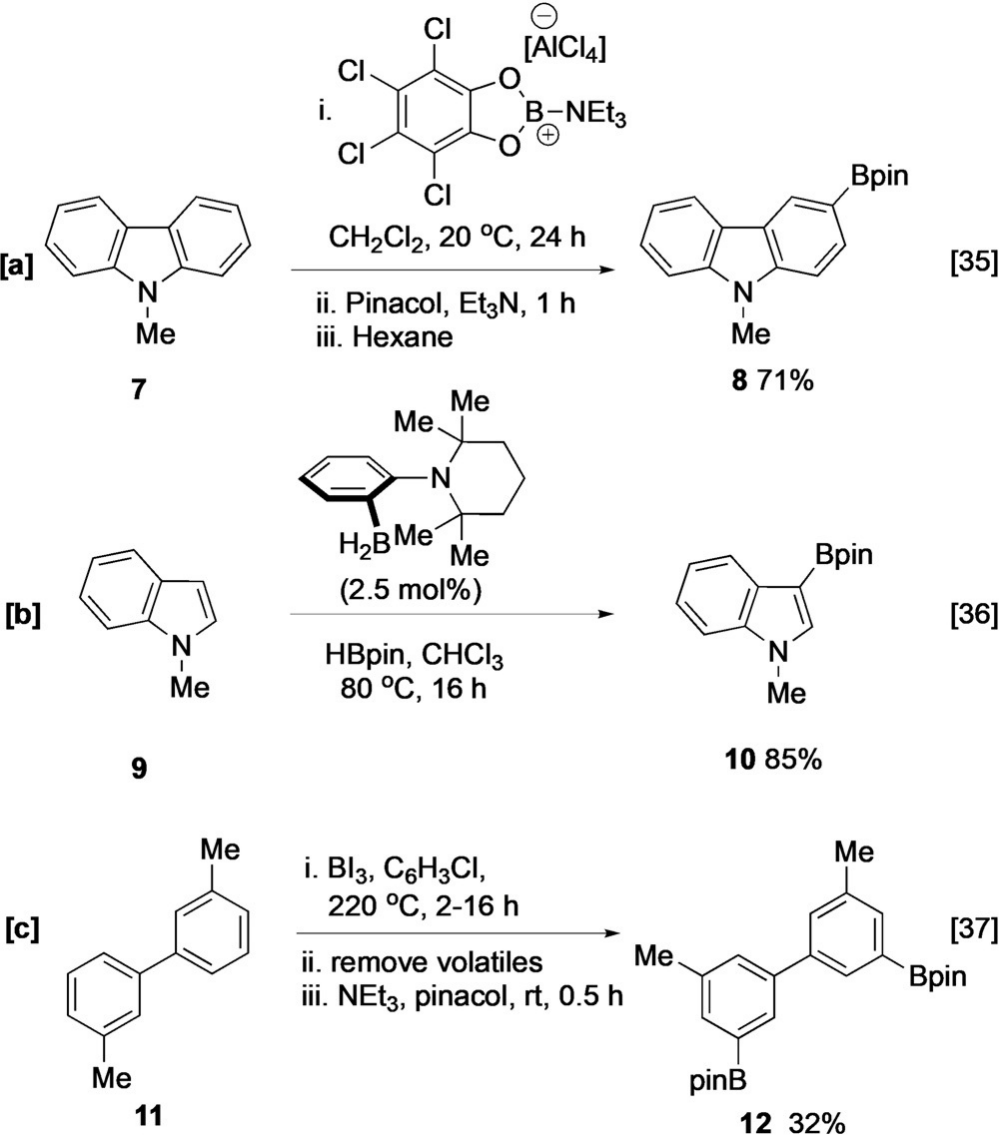
Selected examples of electrophilic aromatic C−H borylation mediated by a) a borenium cation, b) frustrated Lewis pair catalysis, c) BI_3_.

Arene C−H borylation, the direct conversion of a C−H bond to a C−B bond, can be achieved by electrophilic and frustrated Lewis pairs (FLP), or metal‐catalysed pathways. The first of these, involving the reaction of an arene with an in situ generated borenium ion, is generally limited to more nucleophilic arenes, including various heterocyclic systems such as carbazole **7** (Scheme 2 a).^[35]^ Aminoborane frustrated Lewis pairs (FLPs) enable the catalytic dehydrogenative C−H borylation of electron‐rich (hetero)arenes, with similar site‐selectivities (Scheme 2 b).^[36]^ Sterically controlled electrophilic C−H borylation of arenes can also be accomplished using boron triiodide (Scheme 2 c).^[37]^ Much greater substrate scope has been achieved using a number of transition metal catalysts. Of these, iridium trisboryl complexes have become the catalyst system of choice and this review will focus on the application of these systems in the borylation of heterocyclic substrates. Whilst other transition metal complexes including those containing Pd, Co, Fe, Zn, Ru, Ni, Pt, Rh, and Mn also promote similar transformations, these will only be discussed when they offer a distinct advantage in regiocontrol.[^38^, ^39^, ^40^, ^41^, ^42^, ^43^, ^44^, ^45^, ^46^]

### 3.1 Ir‐Catalysed Arene C−H Borylation

**Scheme 3:**
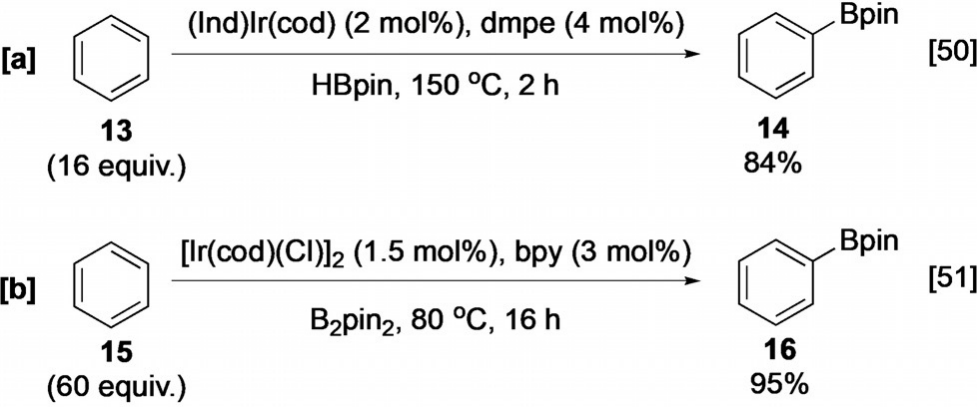
Seminal reports of Ir‐catalysed C−H borylation. bpy=2,2′‐bipyridine, cod=1,5‐cyclooctadiene, dmpe=1,2‐bis(dimethylphosphino)ethane, Ind=indenyl.

Building on earlier work using other iridium boryl complexes,[^47^, ^48^, ^49^] independent publications by Smith (Scheme 3 a) and Hartwig, Ishiyama, and Miyaura (Scheme 3 b) described the catalytic borylation of aryl C−H bonds with phosphine or bipyridine Ir^III^ trisboryl complexes, respectively.[^50^, ^51^] Reflecting higher turnover numbers and more stable catalysts, most C−H borylations are now conducted with variations of the latter system using a combination of [Ir(cod)(OMe)]_2_, 4,4′‐di‐*tert*‐butyl‐2,2′‐bipyridine (dtbpy) or 3,4,7,8‐tetramethyl‐1,10‐phenanthroline (tmphen) as the ligand and B_2_pin_2_ or HBpin as the boron source.[^51^, ^52^, ^53^, ^54^]

**Scheme 4:**
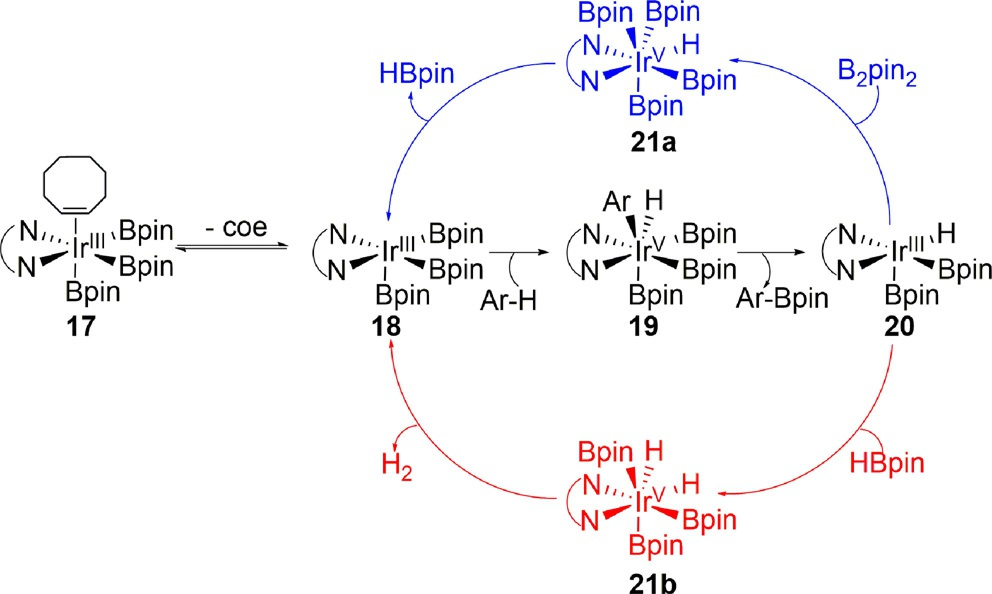
Catalytic cycles of the Ir‐catalysed C−H borylation depicting catalyst regeneration using B_2_pin_2_ (blue) and HBpin (red). coe=cyclooctene.

The generally accepted mechanism involves a catalytic cycle that oscillates between Ir^III^/Ir^V^ intermediates, with the key step involving the activation of the arene C−H bond by the pentacoordinate bipyridyl trisboryl complex **18** (Scheme 4).[^55^, ^56^, ^57^, ^58^, ^59^] Although early computational studies supported the intermediacy of an organoiridium species formed by an oxidative addition pathway, a concerted σ‐bond metathesis pathway influenced by the basicity of the boryl ligands has yet to be ruled out. Consistent with this, calculated transition state energies correlate well with developing negative charge during C−H cleavage at unhindered sites in benzene derivatives.^[60]^ However, Houk et al., in more recent work using distortion/interaction analysis, has demonstrated that a better measure is Ir−C bond strengths which give a robust predictor of regioselectivity.^[61]^ Boryl‐assisted reductive elimination from this highly sterically crowded intermediate **19** produces the aryl boronate and an Ir^III^ bisboryl hydride **20**. The cycle is then completed via the oxidative addition of B_2_pin_2_ or HBpin, followed by the reductive elimination of HBpin or H_2_, respectively, to regenerate **18**. As such, the catalysis can be divided into two distinct cycles according to the boron reagent involved, with B_2_pin_2_ reacting preferentially to HBpin. In general, electron‐deficient arenes are more active than electron‐rich counterparts and the reaction shows good functional group tolerance with a wide range of functional groups being accepted.

**Figure 3:**
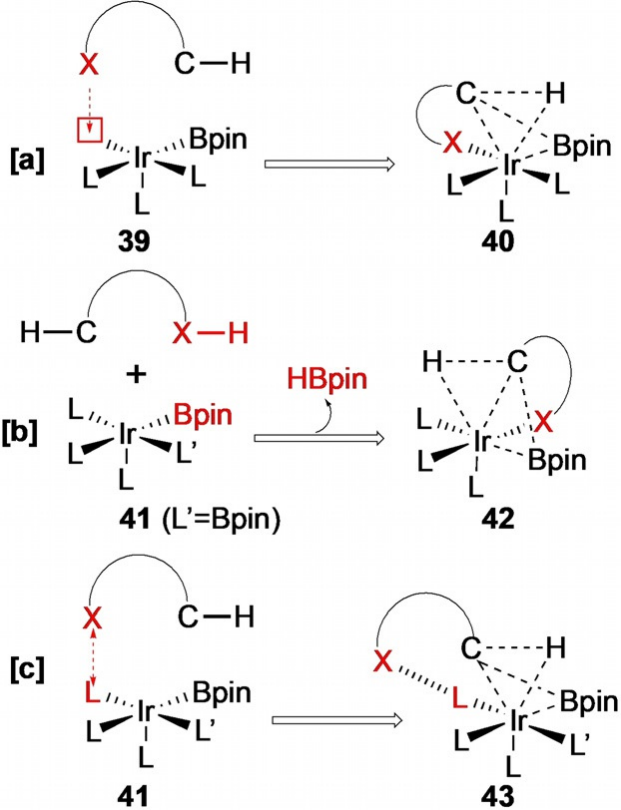
Directed C−H borylation. a) Inner‐sphere, b) relay, and c) outer‐sphere mechanisms.

**Scheme 5:**
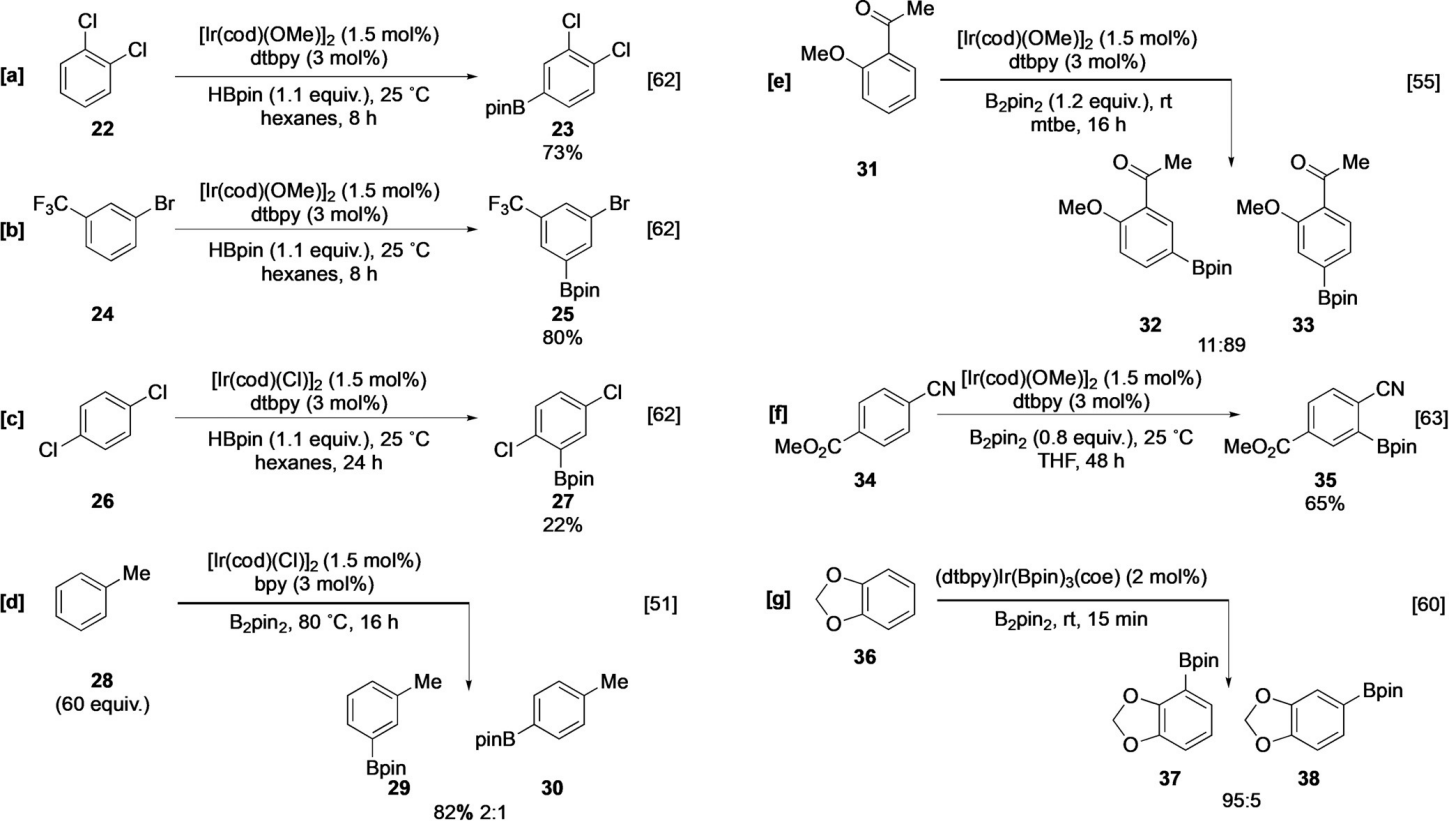
Ir‐catalyzed C−H borylation of arenes. mtbe=methyl *tert*‐butyl ether (IUPAC: 2‐methoxy‐2‐methylpropane).

Due to the sterically crowded nature of the catalytically active species, regioselectivity is generally dominated by steric effects (Scheme 5), with the most accessible positions preferentially activated. The borylation of 1,2‐disubstituted arenes and symmetrical 1,3‐disubstituted arenes, **22** and **24,** proceeds at the uncongested C−H bonds (no *ortho* substituents) affording a single product (Scheme 5 a,b). If the catalyst is not offered an unhindered C−H site, borylation *ortho* to moderately sized substituents can occur, albeit with lower rates and conversions (Scheme 5 c).^[62]^ Substrates with multiple accessible sites give mixtures of products, with monosubstituted arenes such as toluene affording statistical product mixtures at elevated temperatures (Scheme 5 d).^[51]^ At lower temperatures, isomer distributions deviate from sterically determined statistical ratios, alluding to an underlying electronic selectivity. In general, π‐electron acceptors (−M) favour *para* borylation, and π‐donors (+M) (also σ‐acceptors) favour *meta* borylation. (Scheme 5 e).^[55]^ Borylation *ortho* to small strongly electron‐withdrawing substituents (F, CN) is facile, potentially reflecting an electronic activating effect (Scheme 5 f).[^63^, ^64^] Clearer evidence for intrinsic electronic selectivity is seen with benzodioxole **36**, which borylates with near complete selectivity at the more hindered *ortho* position, despite the presence of uncongested C−H sites (Scheme 5 g).^[60]^ This is attributed to the enhanced acidity of these C−H bonds and relates to the intrinsic selectivity observed in many heterocyclic systems discussed below. Reflecting these observations, a major challenge in C−H borylation has been to develop methodologies that afford good levels of control in sterically uncongested substrates, and a number of elegant strategies have been reported which accomplish this. A comprehensive discussion of these is beyond the scope of this article and the interested reader is directed to more specialised reviews.[^65^, ^66^, ^67^] For example, it is possible to use groups within the coordination sphere of the Ir complex to direct the borylation via chelation control. This may be achieved using both inner‐sphere and outer‐sphere directed processes (Figure 3).

**Scheme 6:**
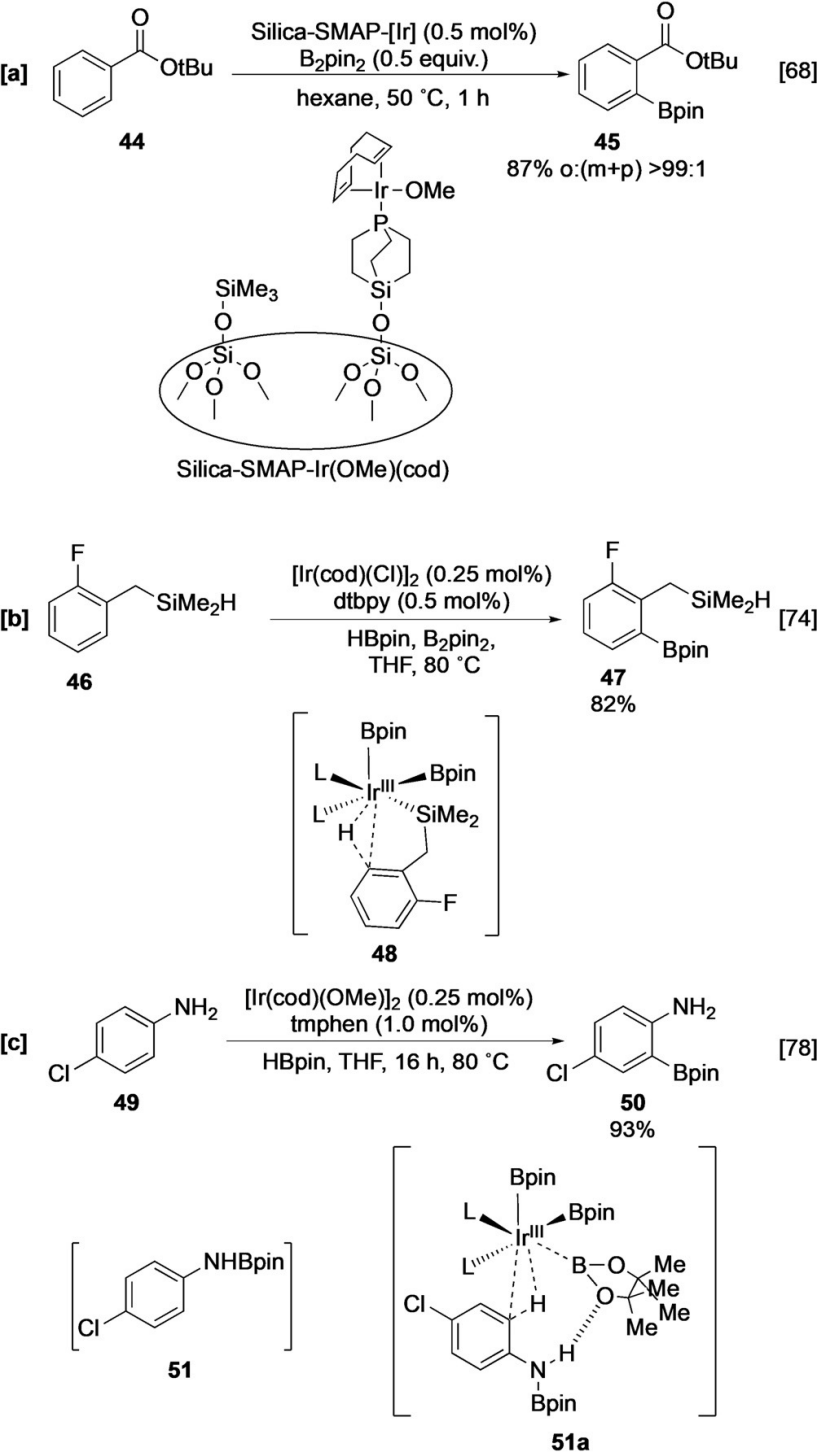
Directed *ortho* borylation by a) inner‐sphere control using Silica‐SMAP, b) relay inner‐sphere borylation, c) outer‐sphere mediated coordination.

Typically, in inner‐sphere directed borylation (Figure 3 a) a substrate containing a ligating element coordinates to the Ir centre, thereby orientating a specific C−H bond for activation. For example, *ortho*‐selective borylation of **44** occurs on reaction with a [Silica‐SMAP]Ir(Bpin)_3_ complex (Scheme 6 a).[^68^, ^69^, ^70^, ^71^] Whilst most of these approaches lead to borylation *ortho* to a coordinating group, more remote C−H activation can occur in relay inner‐sphere borylation (Figure 3 b). In this process, the substrate contains an additional reactive functional group which can ligate the metal centre, displacing one of the boron ligands. As such, binding of the directing group with the metal centre does not necessarily require additional vacant coordination sites.[^72^, ^73^, ^74^, ^75^, ^76^] One such example involves the borylation of hydrosilyl arene **46**, which undergoes selective *ortho* C−H activation following substrate binding to the Ir centre via addition of the Si−H bond (Scheme 6 b). Outer‐sphere directed borylation is a complementary process in which a substrate interaction with a ligand of the catalytically active species leads to regioselective C−H activation (Figure 3 c). For example, an in situ N‐borylated aniline **51** undergoes selective *ortho* C‐borylation facilitated by a hydrogen bond between the aniline N−H and an O atom of the boryl ligand on the active catalyst, as shown in **51 a** (Scheme 6 c).[^77^, ^78^, ^79^] Anilines protected with Boc show similar outer‐sphere directing effects.

**Scheme 7:**
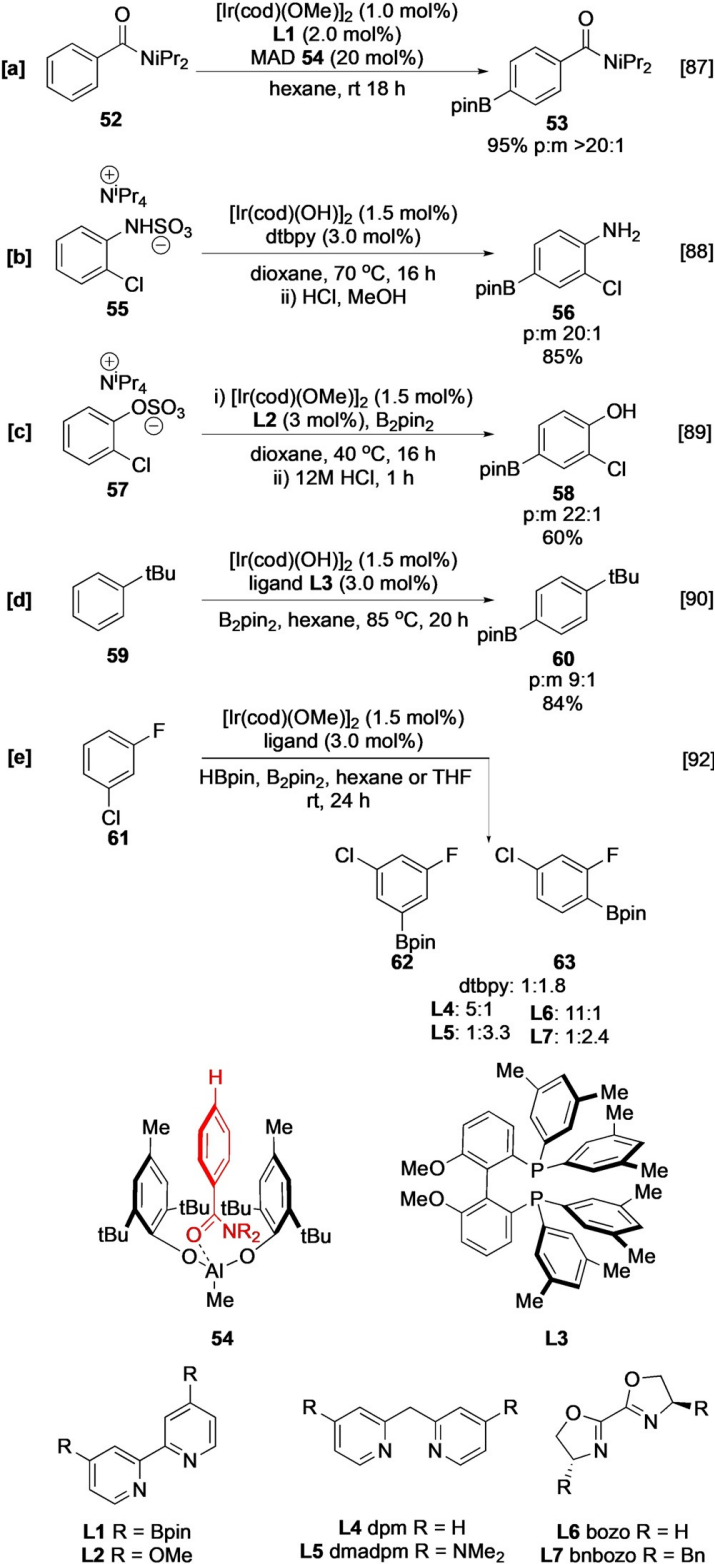
Reagent‐based regiocontrolled C−H borylation.

As with inner‐sphere direction, a variety of ingenious ligands have been described that enable selective borylation at more remote *meta* and *para* positions.[^80^, ^81^, ^82^, ^83^, ^84^, ^85^, ^86^] An alternative strategy has been to design systems that create sterically well‐defined environments that limit accessibility of the substrate to the catalyst (Scheme 7). For example, *para* C−H borylation of aromatic esters and amides can be obtained using cooperative Ir/Al catalysis in which substrate complexation with bulky Lewis acids such as methylaluminum bis(2,6‐di‐*tert*‐butyl‐4‐methylphenoxide) (MAD) **54** limits access to the *ortho* and *meta* positions (Scheme 7 a).^[87]^ Enhanced *para* selectivity is similarly observed in the borylation of sulfamate and sulfate salts in which a tetraalkylammonium counterion shields nominally active *meta* C−H sites (Scheme 7 b,c).[^88^, ^89^] In a ligand‐based approach, the use of the bulky phosphine ligand **L3** creates a flexible reaction pocket at the active catalyst which inhibits the access to *meta* aryl C−H bonds (Scheme 7 d).[^90^, ^91^]

Finally, whilst most ligands are based on a simple bipyridine template, Smith and Maleczka have excitingly shown that alternative motifs have considerable potential to enhance selectivity, with hindered electron‐rich ligands dipyridylmethane (dpm, **L4**) and 4,4′‐bis(dimethylamino)‐2,2′‐dipyridyl)methane (dmadpm, **L5**) favouring steric control, and unhindered electron‐poor ligands 2,2′‐bis‐2‐oxazoline (bozo, **L6**) and 2,2′‐bis[(4*S*)‐4‐benzyl‐2‐oxazoline] (bnbozo, **L7**), favouring greater degrees of electronic control (Scheme 7 e).^[92]^

## 4 Borylation of Heteroarenes

**Scheme 8:**
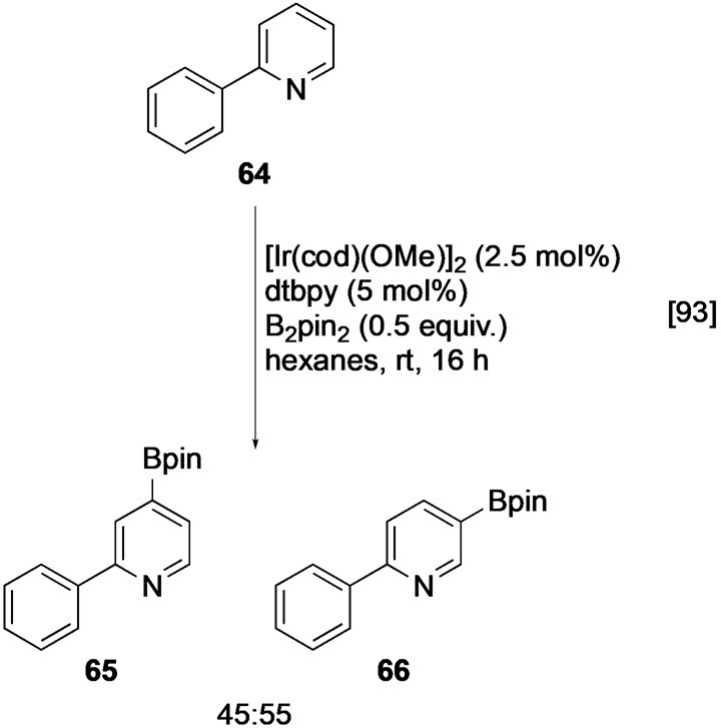
C−H borylation of 2‐phenylpyridine.

The Ir‐catalysed C−H borylation lends itself well to the late‐stage functionalisation of heterocycles because, reflecting the higher C−H acidity of heteroarene C−H bonds, the reactivity of substituted heteroarenes is typically higher than the equivalent benzenoid systems. For example, 2‐phenylpyridine (**64**) is exclusively borylated in the heterocyclic ring (Scheme 8).^[93]^

In contrast to the sterically dominated selectivity observed in carbocyclic arenes, heteroarenes can show a higher degree of intrinsic electronic regiocontrol. It is frequently observed that sterically encumbered C−H bonds can be activated over unencumbered ones, and the position and/or nature of constituent heteroatoms can significantly affect regioselectivity.[^92^, ^94^, ^95^] This review outlines the regioselectivity in the Ir‐catalysed C−H borylation of heteroarenes. It will focus on intrinsic substrate‐based selectivity but highlight examples in which designer catalysts of the types discussed in Section 3.1 have been used to impose reagent‐based regiocontrol. It is organised by substrate classes according to ring system (mono‐, bi‐, polycyclic), ring size (5/6), and number of heteroatoms. Heteroarenes that do not fit into these simple categories are discussed in Section 4.8.

### 4.1 Five‐Membered, Monocyclic, One Heteroatom

#### 4.1.1 Pyrrole, Thiophene, and Furan

**Scheme 9:**
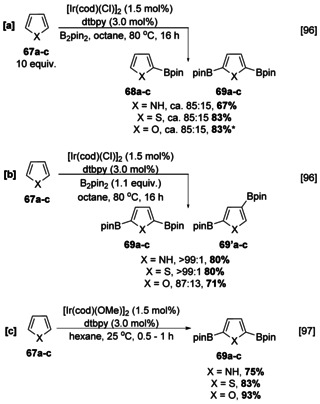
C‐2 selective C−H borylation of pyrrole, thiophene, and furan using a) excess heterocycle (* C‐3‐borylated furan also observed), b) stoichiometric heterocycle, c) room temperature.

Compared to electron‐rich carbocyclic arenes, pyrroles, thiophenes, and furans react much more rapidly and, even in the presence of 10 equivalents of arene, afford a mixture of mono‐ and bisborylated products (Scheme 9 a). Using excess arene leads to higher selectivity for the monoborylated product, but in the case of thiophene this is accompanied by lower efficiency, potentially due to an inhibitory coordinating effect of the sulfur atom on the catalyst. Borylation occurs preferentially at the α position (C‐2 and C‐5) owing to the enhanced C−H acidity associated with the adjacent heteroatom. Reflecting the more pronounced electronegativity of O and hence enhanced reactivity of this heterocycle, lower regioselectivity can be observed in reactions of furans, with small amounts of β‐boryl isomers being detected (Scheme 9 b).^[96]^ All of these heterocycles display high reactivity at room temperature, at which selectivity for furan is improved (Scheme 9 c).^[97]^

**Scheme 10:**
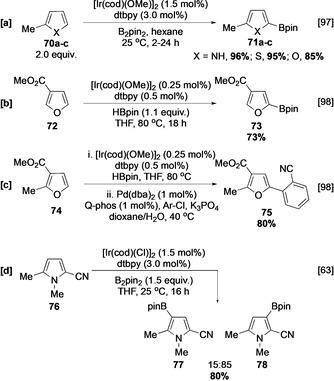
C−H borylation of substituted pyrroles, thiophenes, and furans. dba=dibenzylideneacetone, Q‐phos=1′‐[bis(1,1‐dimethylethyl)phosphino]‐1,2,3,4,5‐pentaphenylferrocene.

Substituents have a similar steric influence as observed in carbocyclic substrates, with reaction occurring preferentially at positions lacking *ortho* substituents.[^63^, ^97^, ^98^] Consequently, 2‐ and 3‐ and 2,3‐substituted heterocycles undergo C−H borylation α to the heteroatom at C‐5 (Scheme 10 a–c). However, reflecting expanded bond angles relative to benzene derivatives, *ortho* substituents are more readily tolerated. For example, some borylation *ortho* to the methyl group in **76** was observed at room temperature, with the major site of C−H activation occurring *ortho* to the nitrile group owing to its lower steric requirement (Scheme 10 d).

**Scheme 11:**
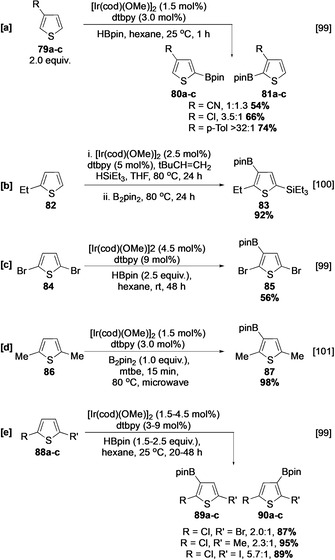
C−H borylation of substituted thiophenes.

The C−H borylation of substituted thiophenes has been thoroughly investigated and these also can undergo reactions at C−H sites that are sterically congested (Scheme 11).^[99]^ For example, the catalyst does not distinguish between the hindered and unhindered α sites in 3‐cyanothiophene (Scheme 11 a). Furthermore, when a tandem C−H silylation/borylation sequence is used, C‐3 borylation of **82** can occur *ortho* to an ethyl group (Scheme 11 b).^[100]^ Given that protodesilylation is straightforward, a sequence involving silylation/borylation/protodesilylation potentially provides a route for formal selective *ortho* borylation of a 2‐substituted thiophene (see also Section 4.4). As with other arenes, reactivity is highly dependent on both the size and electronic nature of the substituents. For example, 2,5‐dibromothiophene (**84**) reacts at room temperature to afford **85** (Scheme 11 c), whereas the more hindered (*A*_Me_≈7 kJ mol^−1^, *A*_Br_≈2 kJ mol^−1^) and electron‐rich dimethylthiophene (**86**) requires more forcing conditions for efficient borylation (Scheme 11 d).[^99^, ^101^] Unsymmetrical 2,5‐disubstituted thiophenes undergo borylation with selectivities that reflect the size of their substituents (Scheme 11 e).

**Scheme 12:**
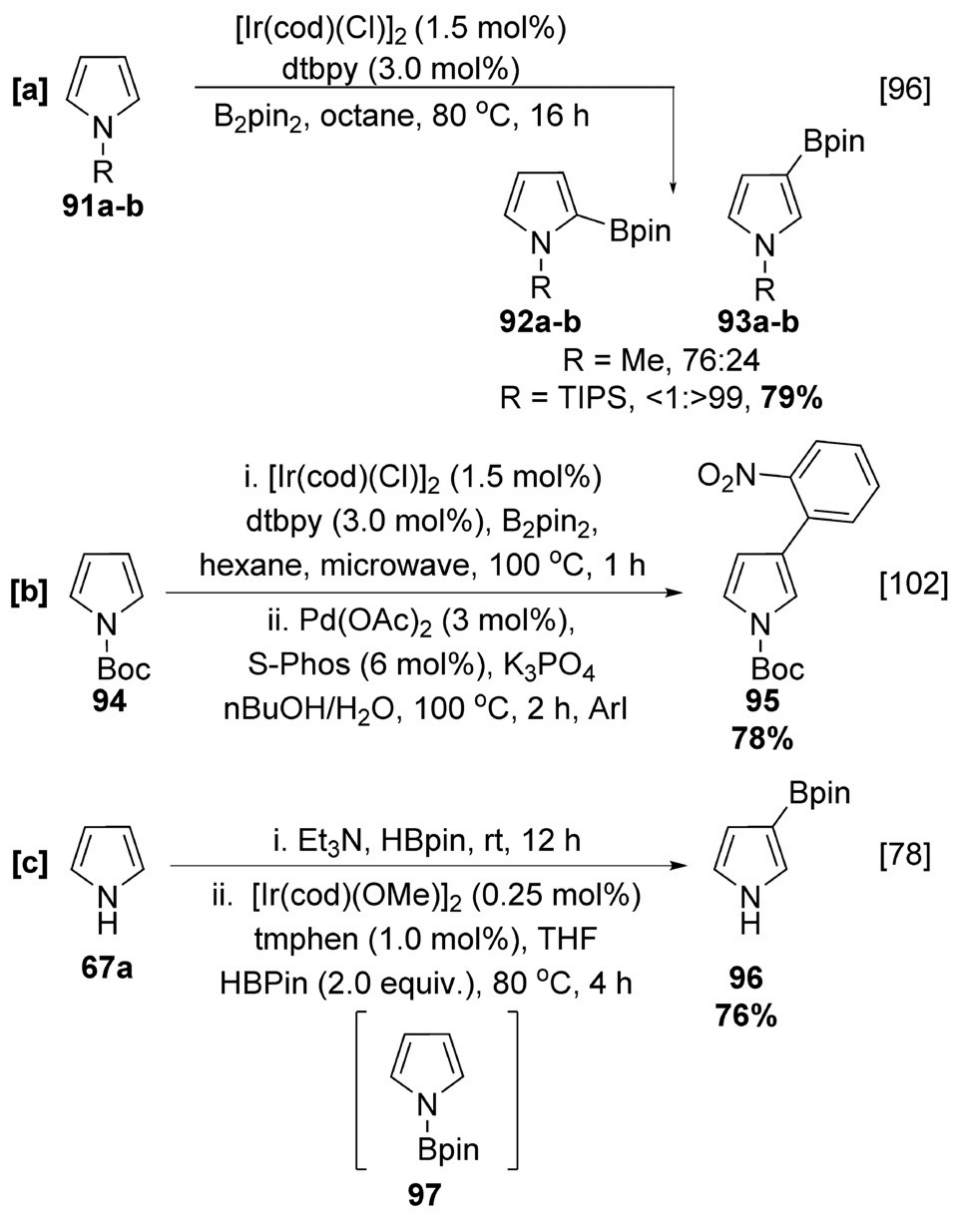
C−H borylation of N‐substituted pyrroles. S‐Phos=2‐dicyclohexylphosphino‐2′,6′‐dimethoxybiphenyl, TIPS=triisopropylsilyl.

Pyrrole is unique in that the N‐substituent can influence the regioselectivity of the borylation reaction. Whilst the parent heterocycle borylates at C‐2, *N*‐methylpyrrole (**91 a**) affords a mixture of the 2‐ and 3‐borylated products in a 76:24 ratio (Scheme 12 a), substrates with larger N‐substituents (TIPS, Boc, Bpin) exclusively give β‐borylated pyrroles (Scheme 12 a,b).[^78^, ^96^, ^102^, ^103^] Whilst TIPS and Boc need to be introduced in a discrete step, Bpin may be installed and removed in situ to provide a “traceless” β‐directed pyrrole borylation (Scheme 12 c).^[78]^

**Scheme 13:**
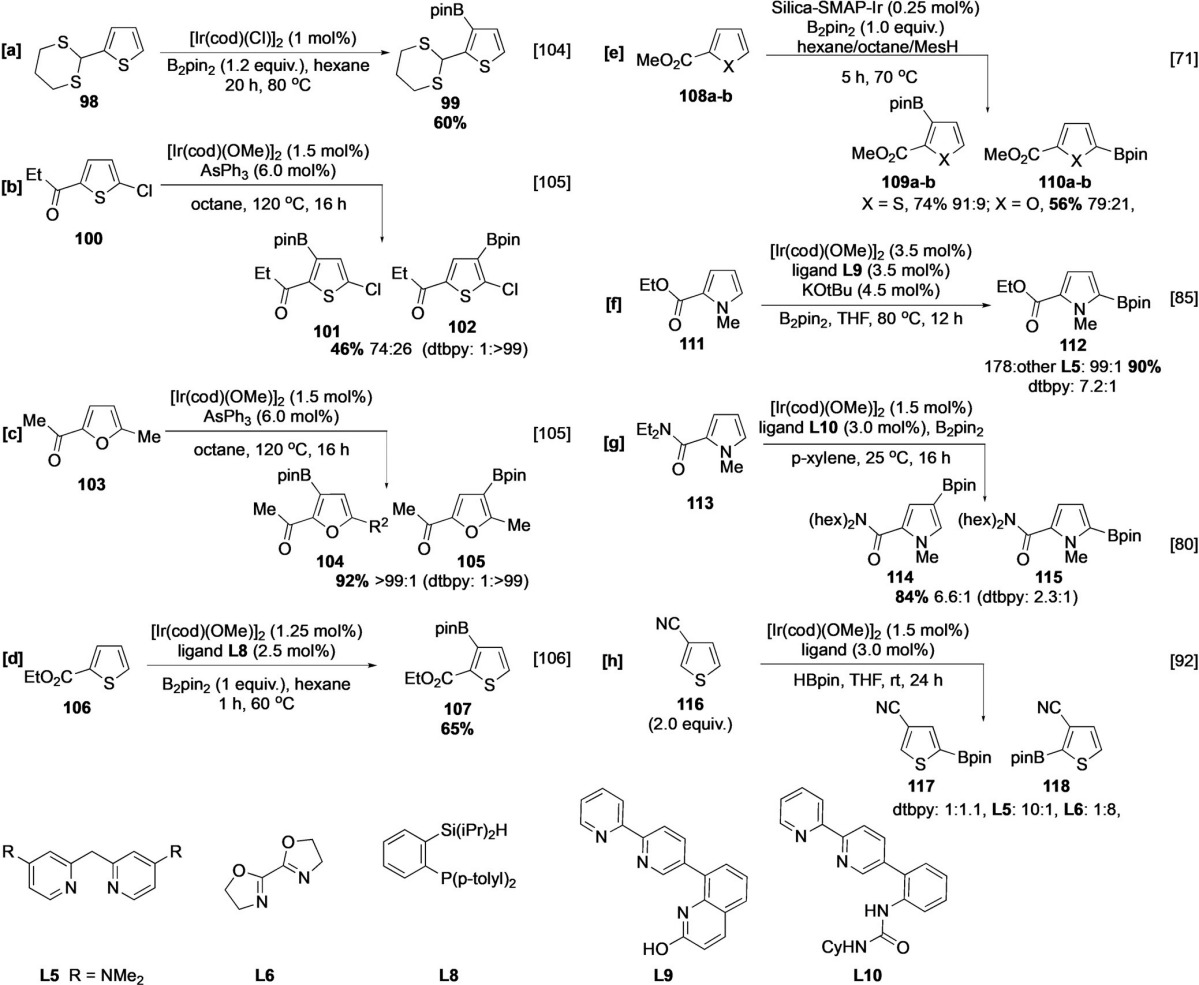
Directed C−H borylation of five‐membered heterocycles. Cy=cyclohexyl.

Alternative C−H borylation selectivities in pyrrole, thiophene, and furan may be obtained using inner‐ and outer‐sphere directing effects (Scheme 13). Most use specifically designed ligands, although a notable exception is the use of the dithiane‐containing substrate **98**. This ligand‐free process affords the 3‐borylated thiophene in the presence of an unhindered α C−H bond (Scheme 13 a), with the dithiane acting as both substrate and ligand.^[104]^ Good levels of *ortho* selectivity in the borylation of pyrrole, thiophene, and furanyl ketones, esters, and amides can be achieved using inner‐sphere directing effects, enabled by specific ligands including AsPh_3_,^[105]^ silyl/phosphorus donor chelates,^[106]^ and Silica‐SMAP,^[71]^ (Scheme 13 b–e). Control experiments indicated that the use of AsPh_3_ provides complementary regioselectivity to that observed with dtbpy, and this method probably relies on the lability of the ligand to produce an open coordination site and enable inner‐sphere direction. This hypothesis is supported by the observation that a chloro substituent, a known inner‐sphere director,^[68]^ leads to lower selectivity, when AsPh_3_ is used, than that observed with the equivalent methyl substituent (Scheme 13 b,c). Similarly, with ligand **L8** a coordinatively unsaturated active catalyst is produced, permitting ligation of ester **106** and facilitating C‐3 borylation in the presence of an otherwise highly reactive α C−H site (Scheme 13 d). With Silica‐SMAP, the directing effect is pronounced enough to facilitate borylation *ortho* to two substituents in the presence of an uncongested α position. However, as above, the enhanced C−H acidity observed in furan ester **108 b** led to some competitive α reactivity (Scheme 13 e).

As with arene borylation, regiocontrol can be attained through the use of designed ligand/additive systems. For example, enhanced 2,5‐selectivity can be observed with the L‐shaped ligand **L9** (Scheme 13 f).^[85]^ Alternatively, hydrogen‐bond‐mediated regiocontrol using urea ligand **L10** improves *meta* selectivity in the borylation of 2‐amidopyrrole **113** (Scheme 13 g).^[80]^ Discrimination between electronic (C‐2) and steric (C‐5) C−H sites can be achieved in 3‐cyanothiophene **116** using bozo **L6** and dmadpm **L5** (Section 1.3.1). In both these last two examples the “standard” dtbpy ligand exhibited poorer selectivity.^[92]^

#### 4.1.2 Porphyrins and Corroles

**Scheme 14:**
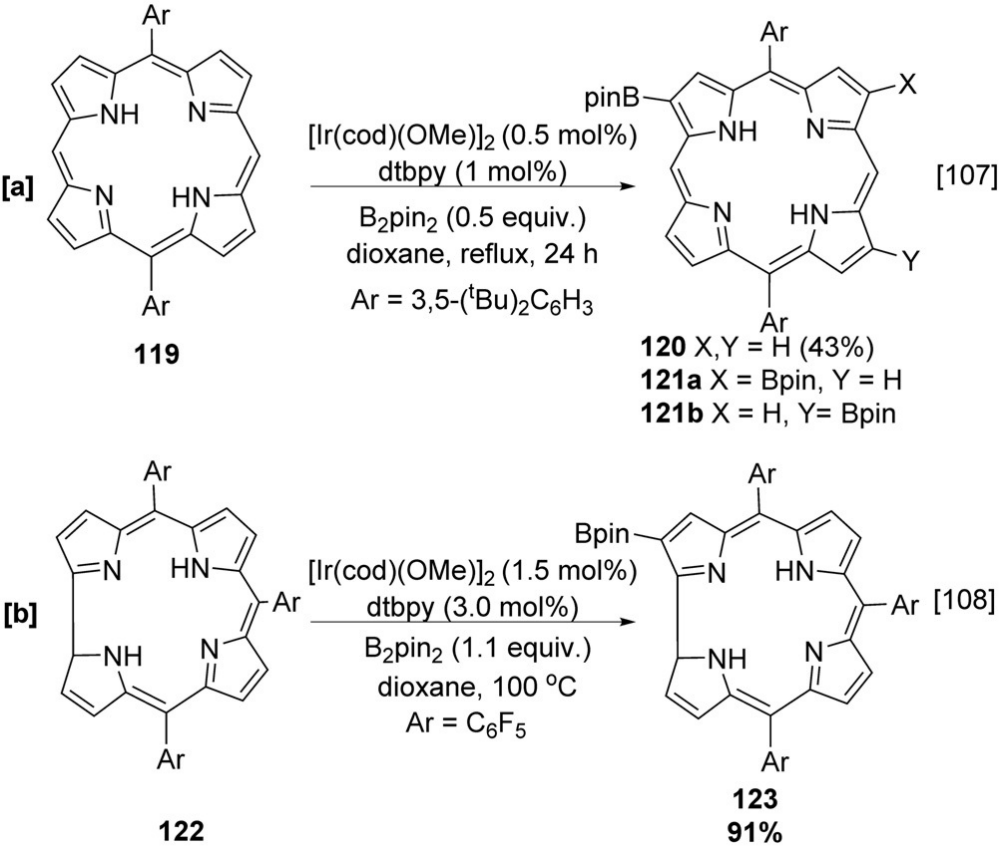
Borylation of porphyrins and corroles.

Porphyrins and corroles are closely related macrocycles consisting of four modified pyrrole units, and therefore they share aspects of borylation regioselectivity with 2,5‐disubstituted pyrroles. For example, the borylation of porphyrin **119**, with limiting boron reagent, occurs at the least hindered pyrrole position minimising *peri* interactions, affording major monoborylated isomer **120 a** alongside two minor bisborylated isomers **120 b** and **120 c** in a 1:1 ratio (Scheme 14 a). Notably, the borylation also tolerates Ni‐ and Cu‐coordinated analogues of **119**, affording products with similar selectivities.^[107]^

Judicious *meso* substitution blocks the corresponding *peri* positions, permitting a degree of regiochemical control in iterative borylation reactions. One borylation event occurs in the reaction of corrole **121** because the other C−H sites are sterically hindered by the *meso* pentafluorophenyl substituents (Scheme 14 b). Substitution at all four *meso* positions in a porphyrin blocks reaction in the macrocyclic ring.[^108^, ^109^]

### 4.2 Five‐Membered, Polycyclic, One Heteroatom

#### 4.2.1 Indole, Carbazole, Benzothiophene, and Benzofuran

**Scheme 15:**
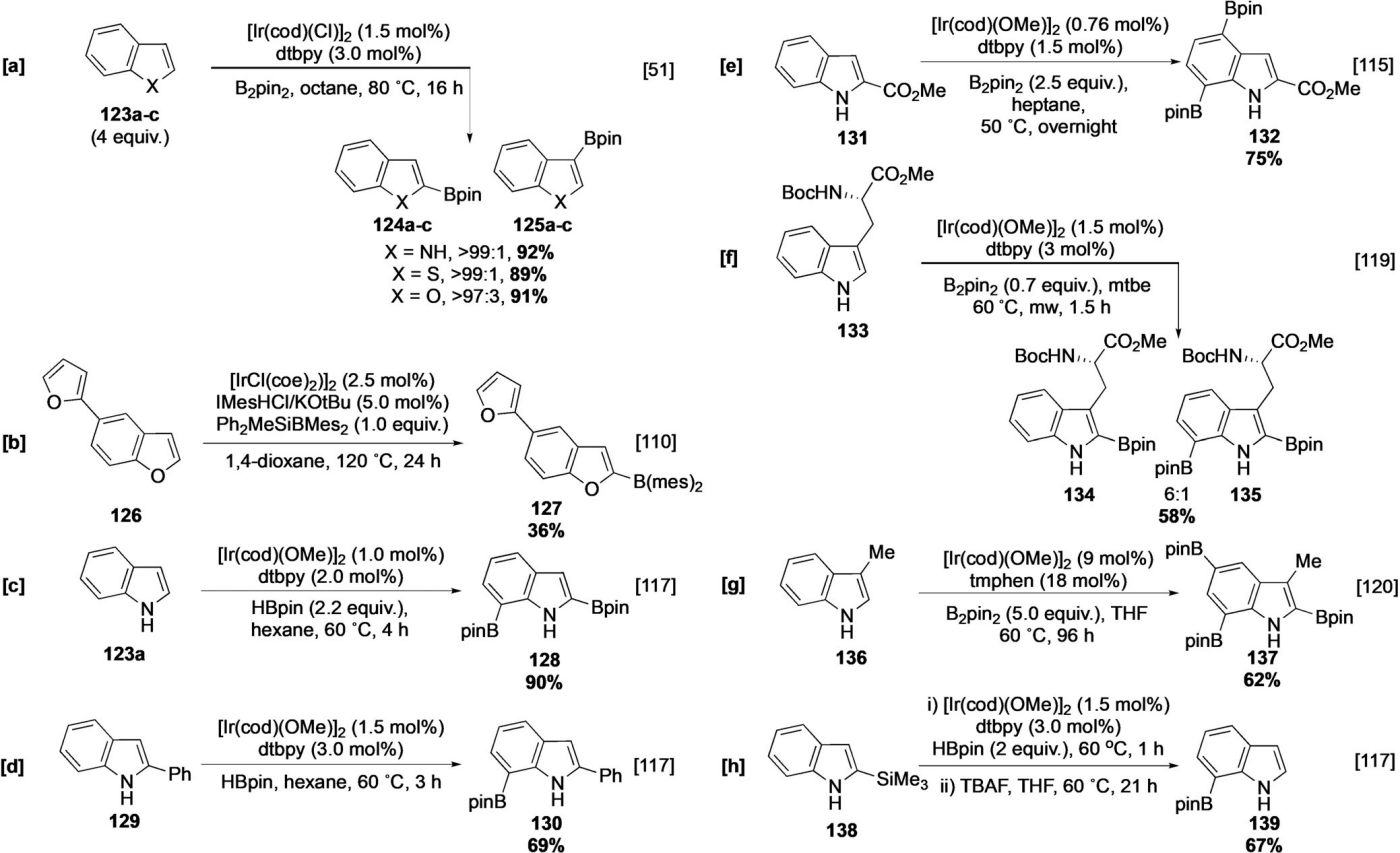
C−H borylation of indoles. IMes=1,3‐bis(2,4,6‐trimethylphenyl)imidazolium.

The presence of a carbocyclic ring in indole, benzothiophene, and benzofuran introduces the potentiality for borylation at multiple sites. However, in all three heterocycles there is a marked preference for borylation in the heterocyclic ring. As with non‐benzofused analogues, the parent heterocycle undergoes borylation selectively α to the heteroatom with excess heteroarene at elevated temperature, with benzofuran **123 c** displaying slightly lower selectivity in analogy to the C−H borylation of furan (Section 4.1; Scheme 15 a).^[51]^ Reducing the arene equivalency and temperature leads to similar product outcomes in these three heterocycles. This contrasts with the electrophilic borylation that occurs in metal‐free systems, which gives the complementary C‐3 functionalised products (Section 3). Interestingly, the borylation of 5‐furylbenzofuran with a silyldimesitylborane reagent occurs selectively at C‐2 of the benzofused heterocycle (Scheme 15 b).^[110]^ Whilst comparison to the traditional C−H borylation systems are challenged by the very different ligand, catalyst and reagents involved, this experiment warrants further investigation into the relative reactivities of benzofused heterocycles and their monocyclic counterparts, and could suggest a higher reactivity of the former ring system.

Owing to the prevalence of indole in pharmaceutical agents, the Ir‐catalysed C−H borylation of many substituted indole derivatives has been well documented and this discussion will focus on this, emphasising differences with the other heterocycles where relevant.[^111^, ^112^, ^113^, ^114^, ^115^, ^116^, ^117^, ^118^] Bisborylation of indole affords the 2,7‐disubstituted product **128**, and other C‐2 substituted indoles, such as 2‐phenylindole, also undergo selective borylation at C‐7. Significantly, reaction of 2‐phenylindole is selective for the fused arene ring, leaving the phenyl substituent intact (Scheme 15 c,d).^[117]^ Polyborylation of 2‐substituted indole **131** occurs initially at C‐7 and then preferentially at C‐4. The latter presumably reflecting the *para* directing effect of a Bpin group (Scheme 15 e).[^63^, ^115^] Reflecting the lower steric demands in a five‐membered ring, 3‐substituted indoles also show good levels of C‐2 selectivity, further emphasising the electronic activating effect of the heteroatom (Scheme 15 f).^[119]^ With higher stoichiometries of B_2_pin_2_, iterative borylation of 3‐substituted indoles such as 3‐methylindole (**136**) can occur, with C−H activation occurring at, successively, C‐2, C‐7, and C‐5 (Scheme 15 g).^[120]^ Formal selective C‐7 mono‐borylation of indole is possible by blocking C‐2 with a labile group, which is subsequently removed. For example, a 2‐silyl substituent can be selectively cleaved using TBAF after having sterically directed borylation to C‐7 (Scheme 15 h).^[117]^

**Scheme 16:**
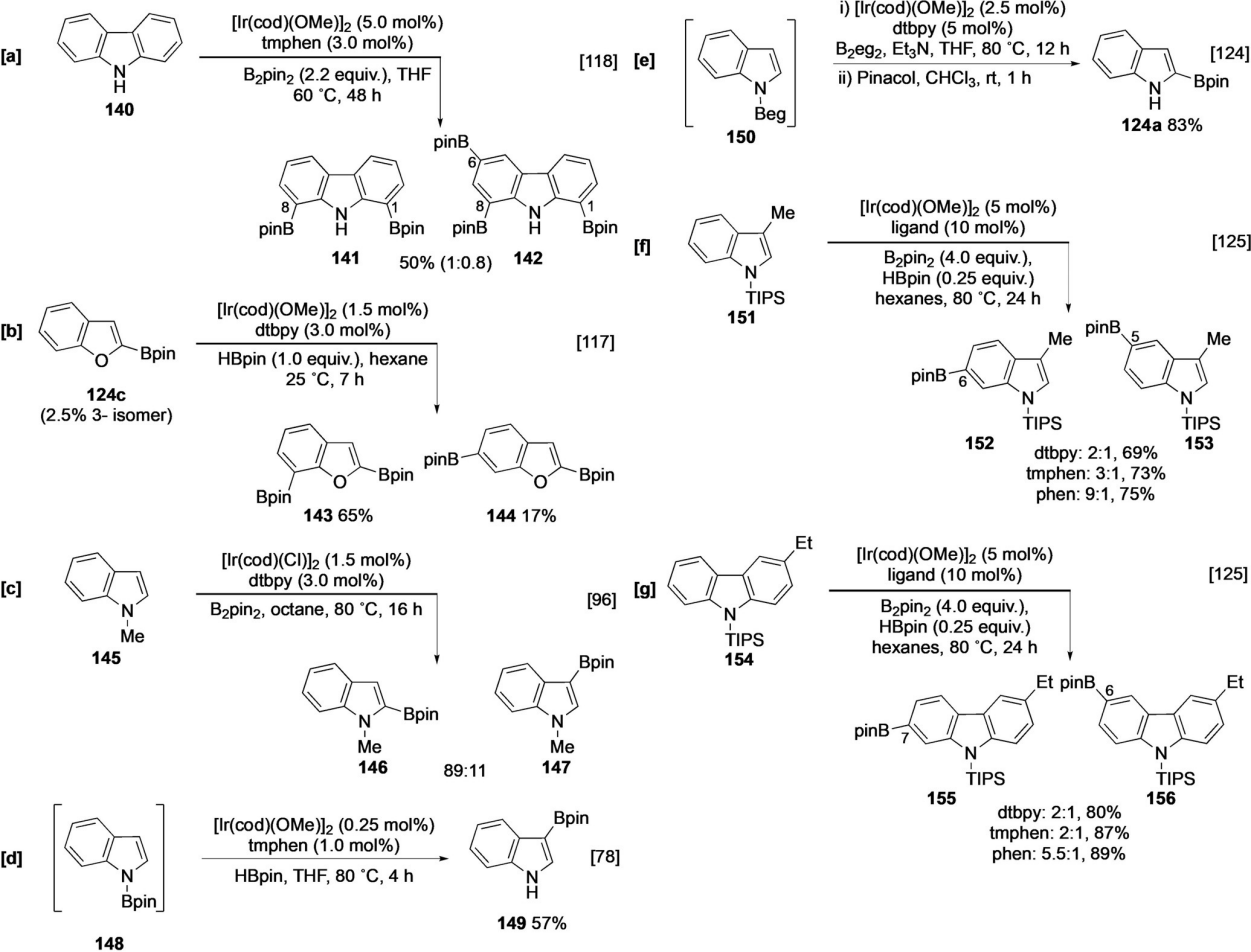
C−H borylation of benzofused heterocycles.

Smith has suggested that the C‐7 selectivity observed with indole originates from substrate ligation to the catalyst, which may promote chelation‐controlled C−H activation. This model can potentially account for the C‐1,8 bisborylation of carbazole **140** (Scheme 16 a). However, the high degree of *peri* steric hindrance at N from the boryl group and the carbocycle might be expected to hinder metal complexation for the second borylation event, suggesting the involvement of an electronic directing effect.^[118]^ Further support for this proposal comes from the borylation of benzofuran, which also shows selectivity for C‐7, albeit with some leakage to the 2,6‐bisborylated product (Scheme 16 b). Whilst the latter observation is consistent with the poorer coordinating nature of the oxygen atom, the possibility for simple intrinsic activation of these positions by the heteroatom is supported by the calculated relative free energies of the anions in 2‐phenylindole (**129**). These indicate that C‐7 (−10.96 kcal mol^−1^) should be the most reactive C−H site when contrasted to other uncongested C−H sites, which range between −9.4 and 0 kcal mol^−1^.^[121]^ Moreover, the selectivities observed with benzofuran are comparable with the *ortho* selectivity observed in the C−H borylation of benzodioxole (**36**; Section 3.1).^[60]^

In analogy to the C−H borylation of pyrrole, the use of N‐substituents can modify the regiochemical outcome, as observed in the borylation of *N*‐methylindole (**145**), which affords a mixture of C‐2 and C‐3 functionalised products (Scheme 16 c). Independent reports give different selectivities for this process and potentially reflect the use of varying solvents, ligands, and reaction times. This collectively suggests that subtly different catalytic cycles/species may exist.[^96^, ^122^] As with their pyrrole analogues, indoles with the bulkier N protecting groups TIPS, Bpin and Boc are borylated with complete β selectivity. The NBpin substrate **148** is prepared in situ in a similar manner to NBpin pyrrole **97** (Section 4.1.1; Scheme 16 d).[^78^, ^96^, ^103^, ^123^] Interestingly, the corresponding reaction with B_2_eg_2_ (eg=ethylene glycolato) affords the α‐borylated product via a process involving an electrostatic outer‐sphere interaction between the NBeg group and the ancillary ligand (Scheme 16 e).^[124]^

If borylation in the heterocyclic ring is sterically inhibited, then borylation in the carbocyclic ring occurs, often with sterically controlled regioselectivity. For example, the borylation of N‐TIPS indole **151** in which C‐2, C‐4, and C‐7 are sterically congested, leads to relatively non‐selective borylation at C‐5 and C‐6. Site‐selectivity can be enhanced by switching the ligand from dtbpy/tmphen to 1,10‐phenanthroline (phen) in indoles and carbazoles, as is observed in the borylation of **151** and **154** (Scheme 16 f,g). This ligand‐mediated selectivity is almost certainly electronically controlled, although further studies are required to determine its origin.^[125]^ Notably, these substrates possess a structure that is comparable to a 1,2‐disubstituted arene, in which electronic effects also contribute significantly to the selectivity observed in reactions run at room temperature.^[55]^

**Scheme 17:**
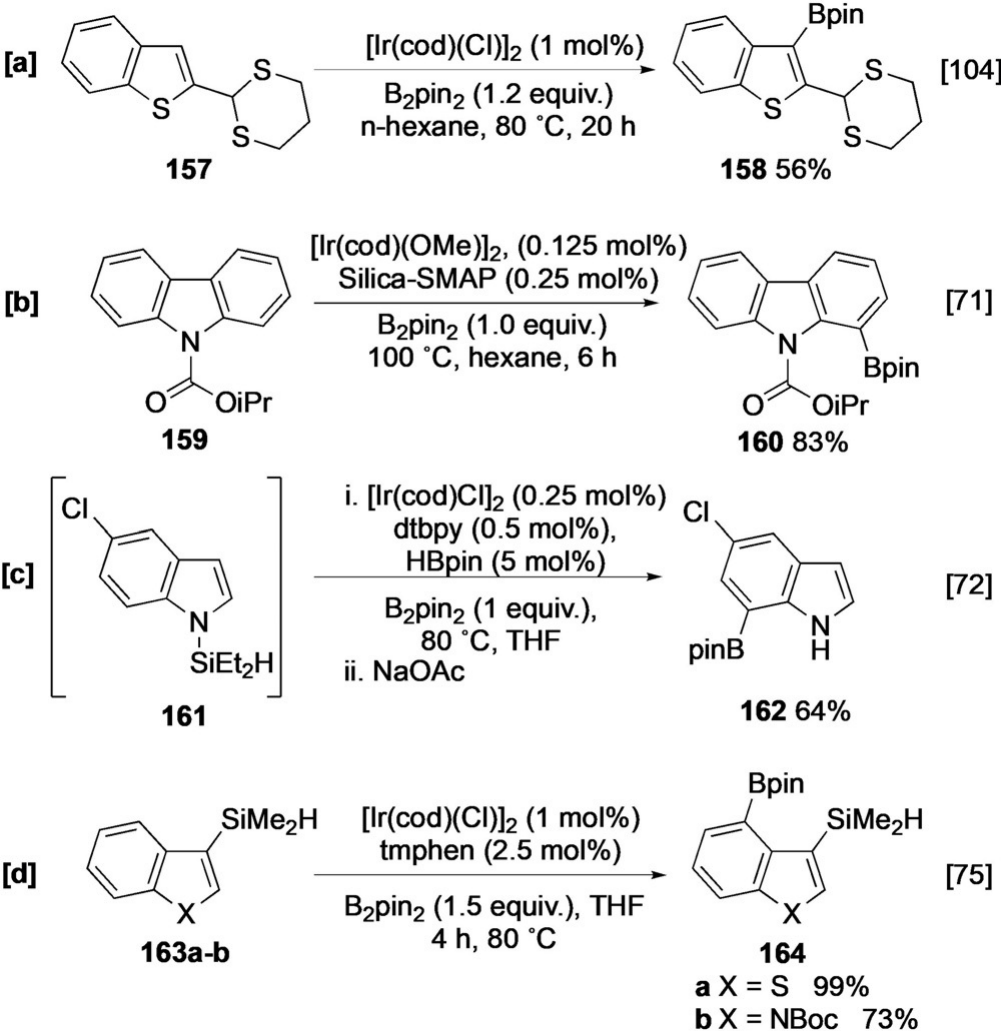
*ortho‐* and *peri‐*directed borylation of benzofused heterocycles.

As observed with their monocyclic equivalents, the intrinsic selectivities of indole, benzothiophene, benzofuran, and carbazoles can be altered using various directing effects. For example, the dithiane‐directed borylation of 2‐substituted benzothiophene **157** leads to an *ortho* functionalised product **158** (Scheme 17 a).^[104]^ Likewise, using Silica‐SMAP, 2‐, 3‐ and N‐substituted carbonyl derivatives of these heterocycles undergo *ortho* or *peri* borylation, as exemplified by the efficient C‐1 borylation of N‐substituted carbazole **159** (Scheme 17 b).^[71]^

Relay direction provides an alternative mode of regiocontrol in polycyclic heterocycles. This can be achieved using a hydrosilyl group that is either generated in situ or pre‐installed. For example, the borylation of chloroindole **161** is completely C‐7 selective (Scheme 17 c), whilst 3‐hydrosilyl benzothiophene and indole **163 a** and **163 b** undergo *peri*‐selective borylation at C‐4 (Scheme 17 d).[^72^, ^75^] In both cases, the bulky nature of the silyl group presumably helps to reduce competing C‐2 borylation.

**Scheme 18:**
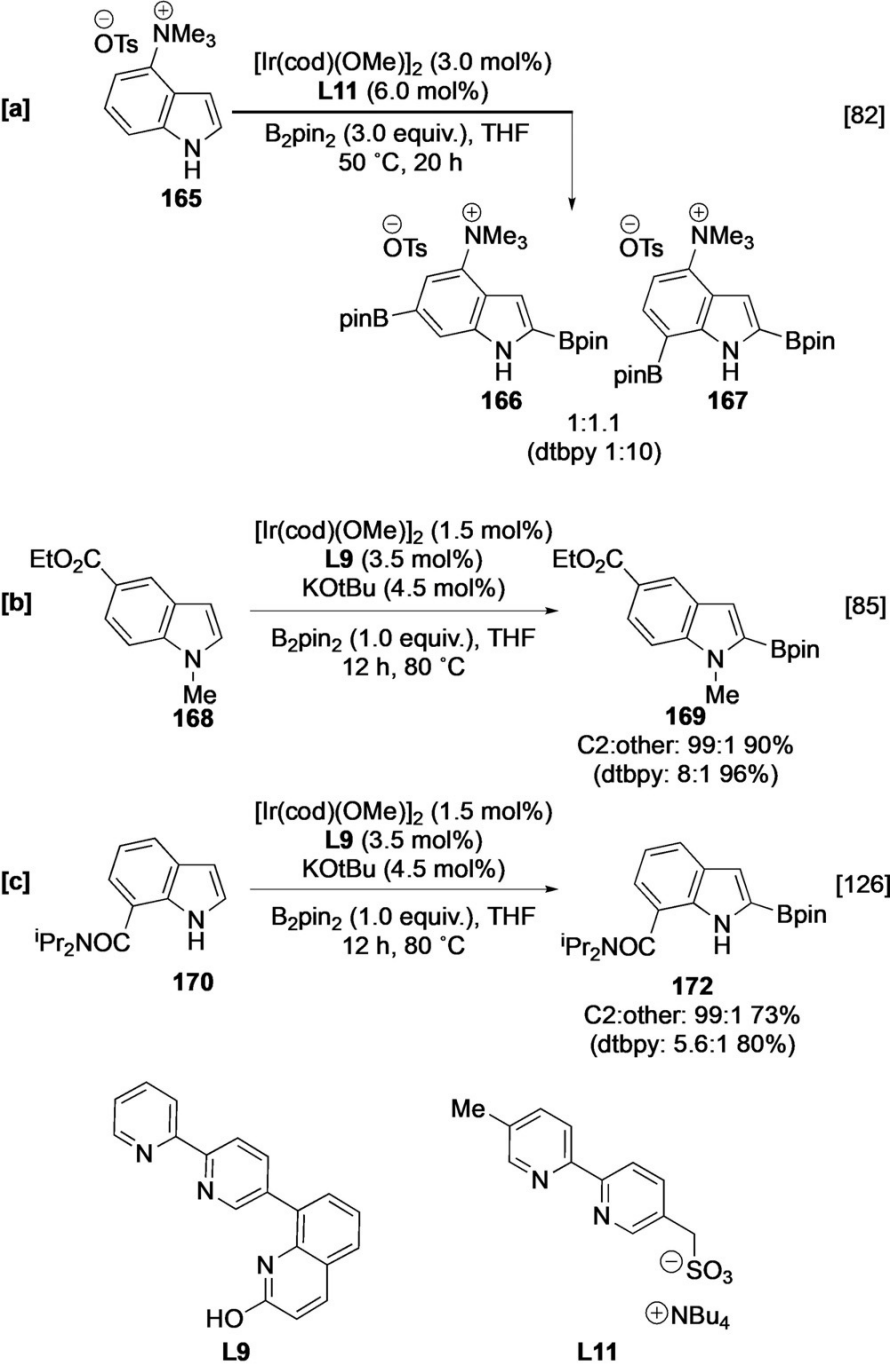
Inner‐sphere directed borylation of indoles.

Outer‐sphere systems can also alter the selectivity in substituted indole derivatives. For example, ion‐pair recognition enables C‐6 *meta* borylation to compete with C‐7 functionalisation of ammonium indole **165** using ligand **L11** (Scheme 18 a),^[82]^ whilst C−H borylation with L‐shaped ligand **L9** leads to enhanced α selectivity in indole ester **168** and indole amide **170** (Scheme 18 b,c).[^85^, ^126^]

#### 4.2.2 Indazoline

**Scheme 19:**
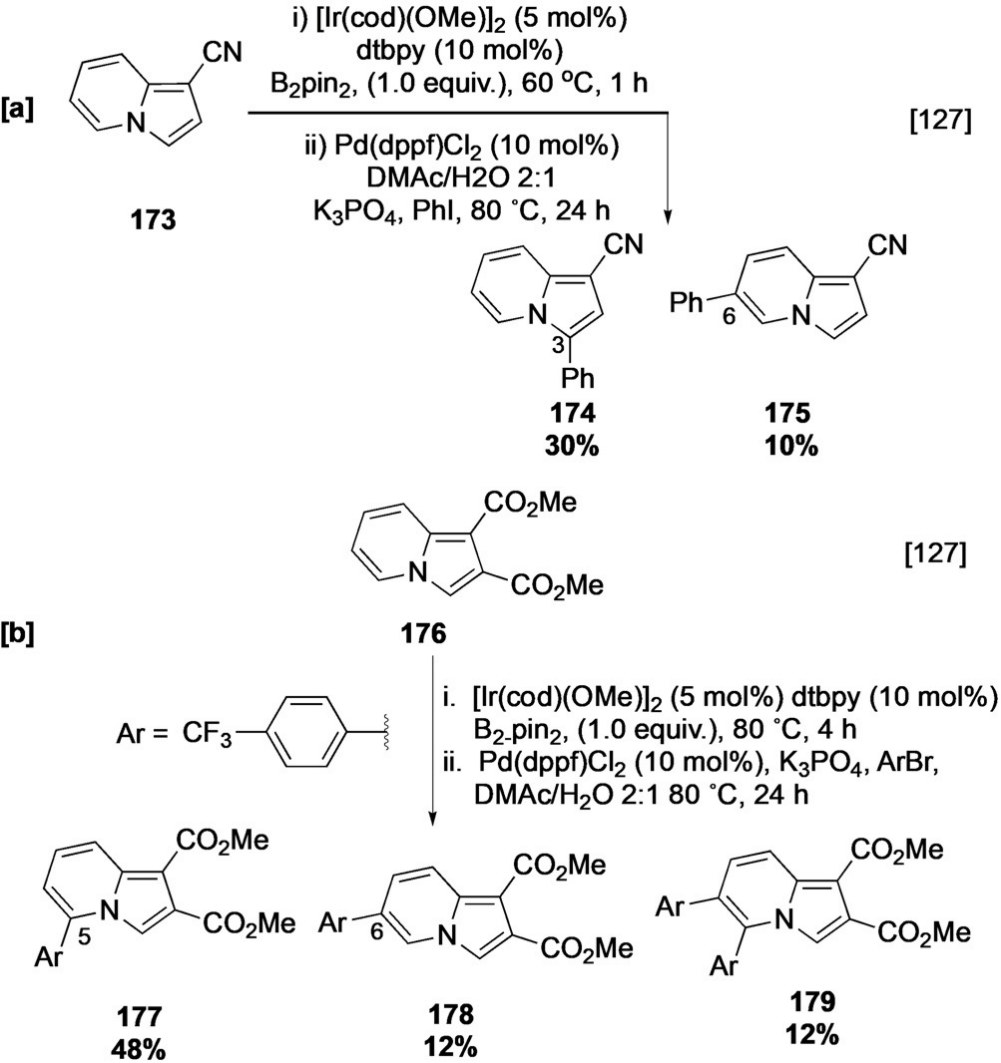
C−H borylation of indazoline derivatives. DMAc=dimethylacetamide.

Indazoline is structurally related to indole and may be employed as a bioisostere. Whilst the C−H borylation of the parent heterocycle is slow, substitution with electron‐withdrawing groups enhances reactivity.^[127]^ In analogy to the C−H borylation of pyrrole, cyanoindazoline **173** undergoes C‐3 borylation α to the heteroatom, and additionally displays reactivity at C‐6, and this is similarly observed for indazoline diester **176** (Scheme 19). Curiously, in this latter transformation the 5,6‐bisarylated **179** was also produced, indicative of an unusual C−H borylation occurring *ortho* to a Bpin substituent.

### 4.3 Six‐Membered Monocyclic Heterocycles with One Heteroatom

#### 4.3.1 Introduction

Heteroarenes that contain basic azinyl nitrogen atoms represent a distinctive challenge for borylation chemistry. In contrast to the selective α borylation observed in azole N‐containing rings, borylation is electronically disfavoured α to an azinyl N atom. This can crudely be likened to the steric inhibitory effect of a substituent but is best attributed to dipolar repulsion between the azinyl lone pair and the developing negative charge on the α carbon atom in the C−H activation transition state. In a given substrate, the degree to which α‐azinyl borylation occurs is dependent on the electron density at N, and on steric constraints in the rest of the molecule. DFT calculations of the reaction pathway for the C‐2 borylation of pyridine show ca. 1 kcal mol^−1^ higher barrier compared to borylation at the other sites.^[128]^ As this is a relatively small difference, the lack of α‐azinyl products can also be attributed to the poor stability of 2‐azaarylboronates. These are known to decompose via several pathways, including protodeborylation.^[129]^ However, the introduction of a boryl group in the α‐azinyl position may be promoted by stabilising groups, and these are typically electron‐withdrawing groups such as other N ring atoms, sulfonyl or trifluoromethyl groups, and halides.

#### 4.3.2 Pyridine

**Scheme 20:**
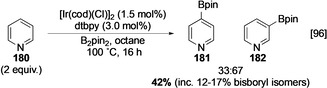
Ir‐catalysed C−H borylation of pyridine.

**Scheme 21:**
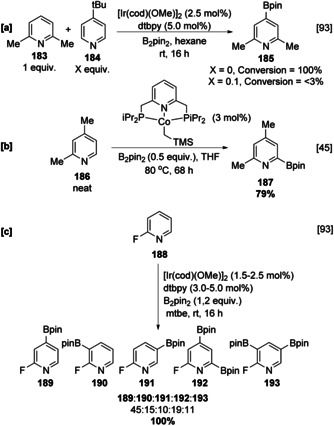
C−H borylation of 2‐substituted pyridines. TMS=trimethylsilyl.

**Scheme 22:**
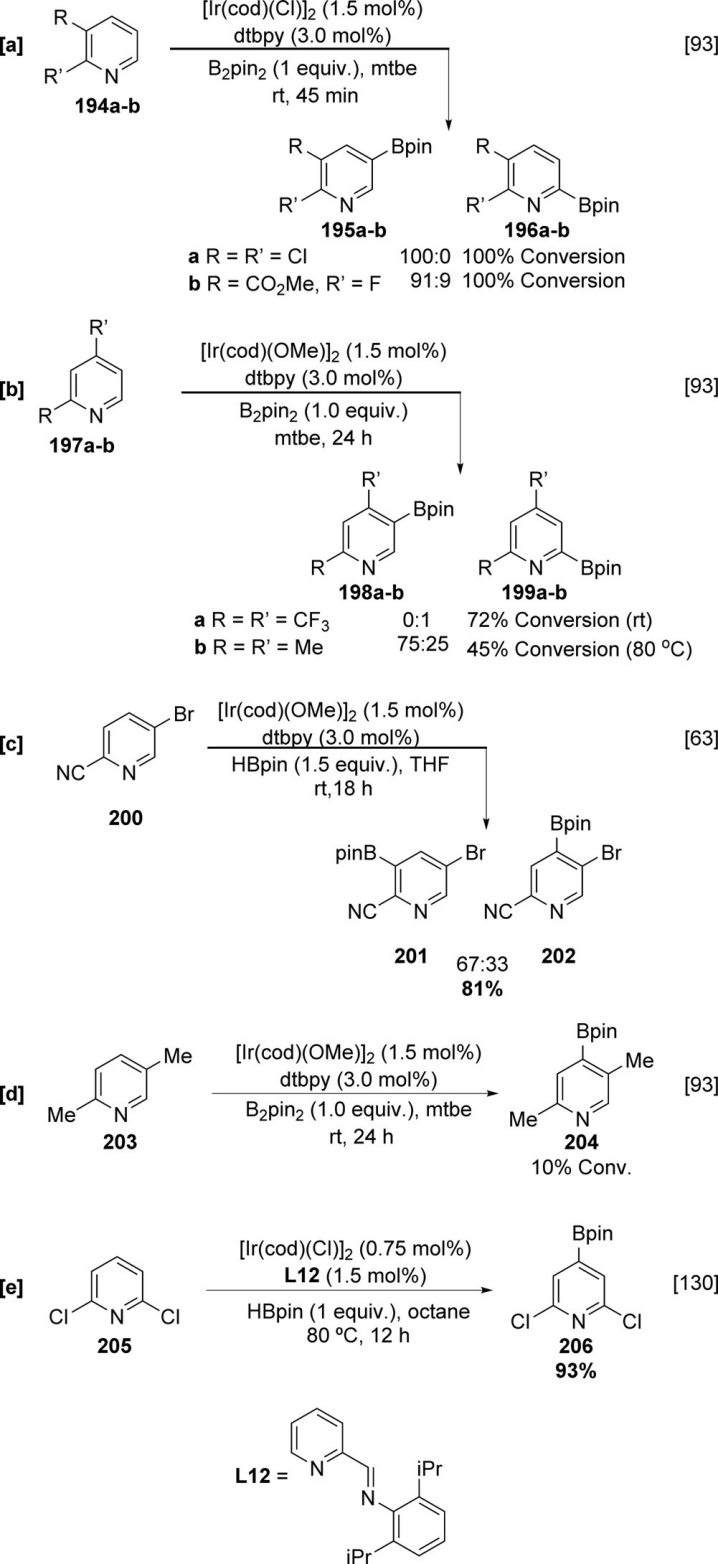
C−H borylation of substituted pyridines.

In the original report on the Ir‐catalysed C−H borylation of heteroarenes, pyridine **180** stands out as an unusually inactive substrate, requiring increased temperature and affording a statistical mixture of C‐3 and C‐4 borylated products in low yield (Scheme 20).^[96]^ C‐2 borylated products were not observed, and this is due to the inhibitory effect of the azinyl nitrogen. In contrast, borylation of various 2‐substituted pyridines occurs readily at room temperature with the expected sterically controlled selectivity (Scheme 21). The complete selectivity for reaction in the heterocyclic ring observed with 2‐phenyl pyridine provides further illustration of the higher reactivity of heterocyclic vs. carbocyclic arenes. Although the low yields in the borylation of pyridine may be attributed to the rapid decomposition of in situ C‐2 borylated products, a more likely explanation is the reversible inhibition of the active catalyst through substrate ligation. Evidence for this is seen in the relative borylation of 2,6‐lutidine (**183)** and 4‐*tert*‐butylpyridine (**184**). The latter, lacking unhindered non‐azinyl C−H bonds, is inert, whilst the former undergoes facile C−H borylation selectively at the sterically uninhibited 4‐position (Scheme 21 a). However, the addition of small amounts of **184** into the borylation reaction of **183** efficiently inhibits the reaction through coordination to the vacant site on the catalytically active iridium trisboryl complex.^[93]^ Whilst sterically blocked substrates such as 2,4‐disubstituted pyridines generally display poor reactivity, Chirik's pincer‐ligated cobalt complexes can smoothly promote C−H borylation of 2,4‐lutidine (**186**) α to the azinyl nitrogen, with C‐2 functionalised pyridine **187** being detectable in 79 % GCMS yield (Scheme 21 b).^[45]^ It is more difficult to activate this position using Ir (see Scheme 22 b), and this likely suggests that a different mechanistic pathway is operating with this Co‐catalyst. In the Ir‐catalysed C−H borylation of azinyl heterocycles, the impact of substrate inhibition can be reduced by an electronegative substituent that lowers the basicity of the azinyl nitrogen, weakening the interaction with the catalyst. For example, the C−H borylation of 2‐fluoropyridine is facile at room temperature and gives rise to five isomeric pyridyl boronates. Electron density is reduced at N by F to a sufficient extent such that the α‐azinyl borylated product **192** is observable (Scheme 21 c).

Other substituted pyridines show a selectivity that is a balance of sterics and electronics. For example, 2,3‐disubstituted pyridines borylate largely with steric control at C‐5 but more strongly electron‐deficient systems display enhanced reactivity at the α azinyl position (Scheme 22 a). Similar trends are observed with 2,4‐disubstituted pyridines, with the inhibitory effect of the azinyl nitrogen leading to borylation occurring at C‐5 providing that the bulk of the C‐4 substituent can be tolerated. As with **188**, pyridine **197 a** is sufficiently electron‐deficient and C−H borylation is selective for C‐2 even at room temperature. In contrast, electron‐rich and sterically congested pyridine **197 b** requires more forcing conditions and is selective for C‐5 (Scheme 22 b).

**Scheme 23:**
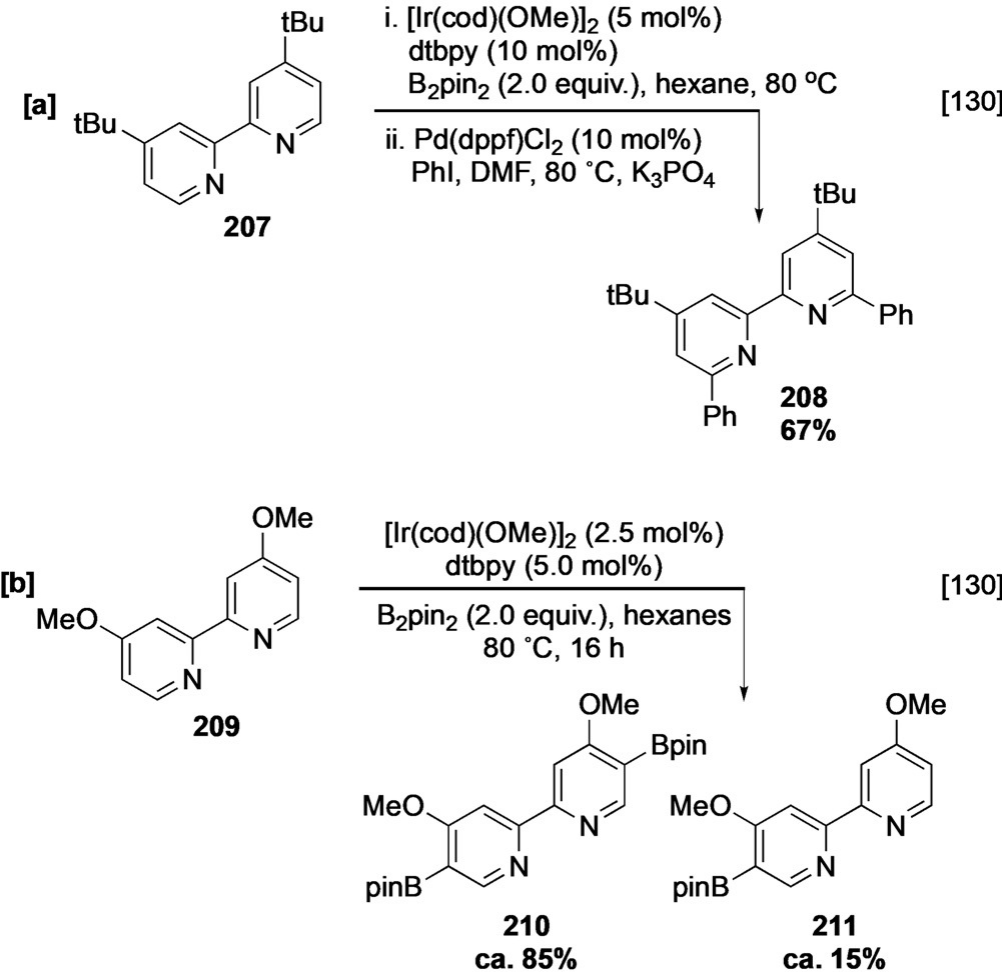
Borylation of 2,2‐bipyridines.

Interestingly, dtbpy **207** undergoes C−H borylation under more forcing conditions at the α azinyl positions, although this was not observed in the absence of excess dtbpy, suggesting that ligand dissociation does not readily occur during catalysis (Scheme 23 a).[^53^, ^130^] The regioselectivity was confirmed using a one‐pot Suzuki–Miyaura cross‐coupling to deliver arylated product **208** and this ability to directly use borylated products is a valuable strategy for these less stable boronate esters. Given that the related 4‐*tert*‐butylpyridine **184** is unreactive (vide supra), the 2‐pyridyl unit in **207** likely facilitates activity by functioning both as a steric blocker and an electron‐withdrawing (activating) group. Steric effects remain the dominating influence in this transformation, as the corresponding 4,4′‐dimethoxybipyridine analogue **209** gives the *ortho* methoxy functionalised products **210** and **211** (Scheme 23 b), in which borylation occurs remote to both the azinyl nitrogen and the pyridyl substituent. Borylation of 2,5‐disubstituted pyridines is only viable with moderately sized substituents and, given the inhibitory effect of the azinyl nitrogen, will occur preferentially at C‐3 or C‐4 in a ratio that reflects the competing steric and electronic influences of the substituents. For example, the borylation of 2‐cyano‐5‐bromopyridine (**200**) proceeds efficiently to afford C‐3 and C‐4 boronates **201** and **202** in a 2:1 ratio (Scheme 22 c),^[63]^ whilst 2,5‐lutidine (**203**) shows poor conversion under comparable conditions (Scheme 22 d). Finally, 2,6‐disubstituted pyridines, for which the inhibitory effect of the azinyl nitrogen is blocked, react as electron‐deficient arenes and generally have good activities exhibiting C‐4 selectivity, and ligands based on the bipyridine scaffold such as **L12** also promote this transformation (Scheme 22 e).^[131]^

**Scheme 24:**
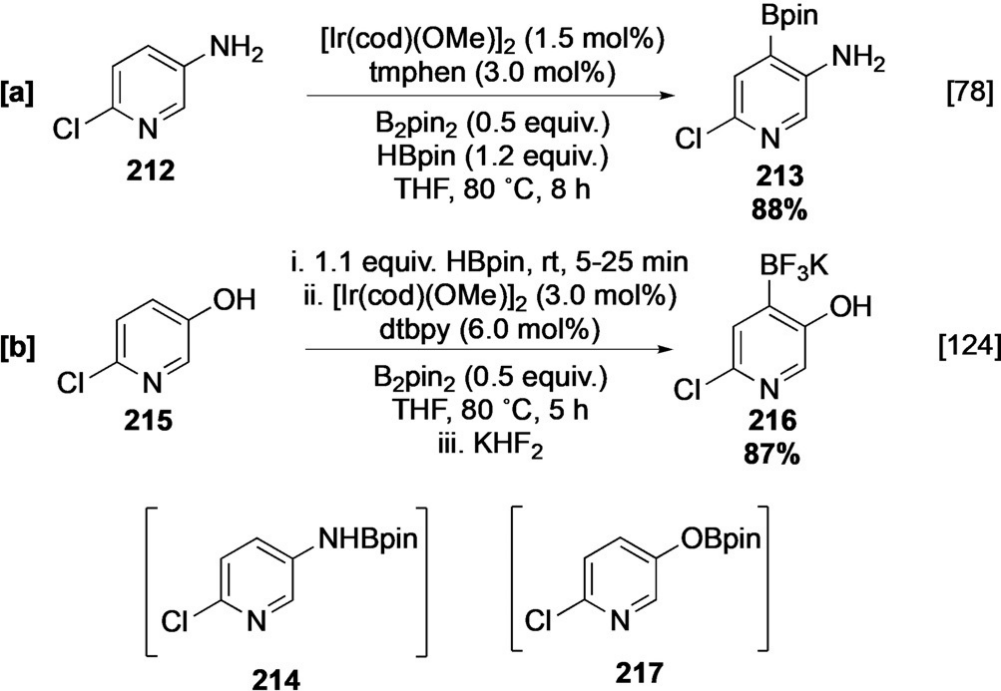
Outer‐sphere directed C−H borylation of pyridines.

**Scheme 25:**
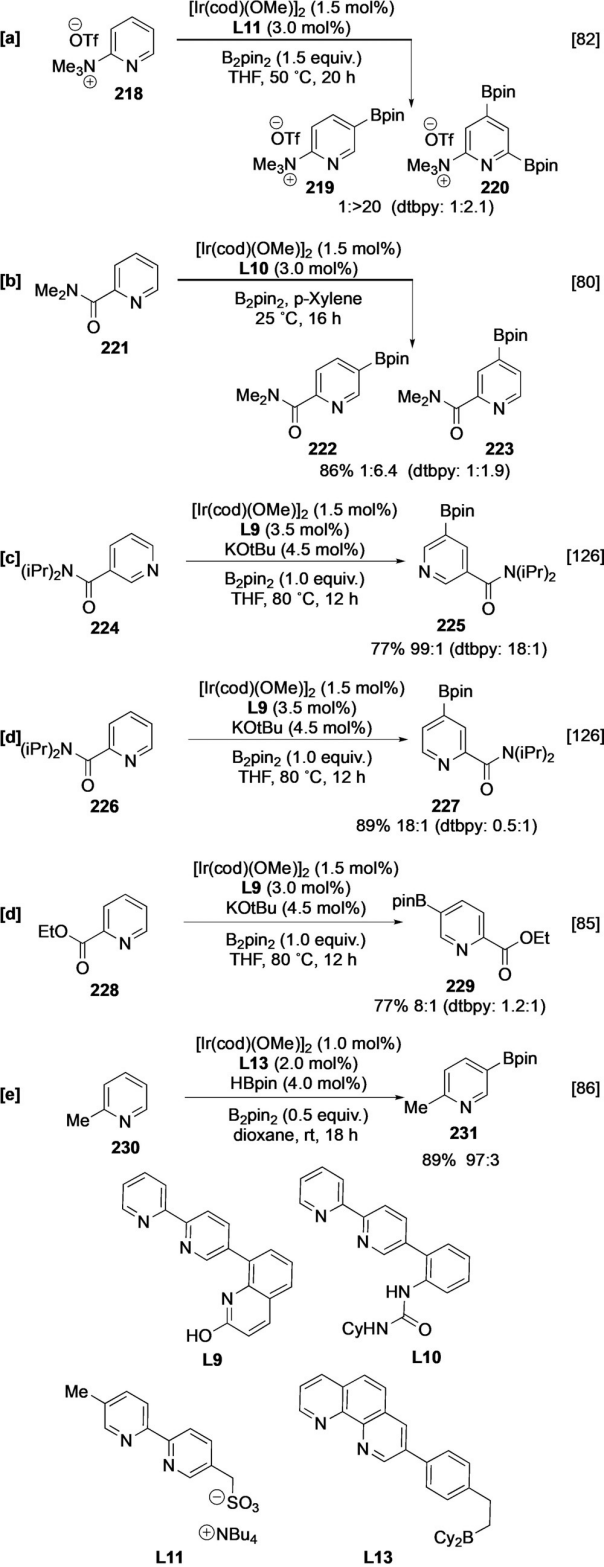
Ligand‐mediated outer‐sphere directed C−H borylation of pyridines. OTf^−^=triflate.

Outer‐sphere directing effects have been exploited to override these selectivities. For instance, aminopyridines undergo rapid NH borylation to form the corresponding NHBpin adduct. This intermediate facilitates *ortho‐*selective borylation in 2‐substituted 4‐ and 5‐aminopyridines, and this may be seen in the borylation of **212**, via the initial formation of **214** in situ (Scheme 24 a). In addition, the borylation of 5‐hydroxypyridine (**215**) is *ortho* (to OH) selective following traceless O‐borylation with HBpin to afford C‐4 functionalised **216** (Scheme 24 b).[^78^, ^124^] However, it is unclear to what extent the regiochemical outcomes of these processes differ from intrinsic regioselectivity. In particular, it can be argued that, given the relatively similar size of each substituent, the selectivity is a measure of *ortho* activation due the competing electron‐withdrawing effect of the nitrogen and oxygen substituents, respectively. In support of this, traceless *ortho* direction is not displayed with 2,6‐aminopyridine derivatives, and sterically mediated C‐4 borylation analogous to the borylation of **205** occurs instead.^[78]^ Other outer‐sphere (ligand‐mediated) directing systems can also influence the regioselectivity of the borylation of pyridines. For example, when ionic ligand **L11** is employed, the C‐4 selectivity in pyridyl amides and trialkylammoniums is increased, and this can be observed in the borylation of **218**.[^82^, ^83^, ^84^] Following C‐4 borylation, the powerful electron‐withdrawing capacity of the trimethylammonium group can facilitate borylation at C‐6, affording bisborylated **220** selectively (Scheme 25 a). Alternative selectivities can also be obtained using ligand‐based complexation to direct borylation of pyridine amides and esters (Scheme 25 b–e).[^80^, ^85^, ^126^] Control experiments using dtbpy indicate that the C‐2 substituents in each substrate possess modulating effects on intrinsic site selectivities which deviate from simple steric control. Notably, the complexation of isonicotinamide **224** to L‐shaped ligand **L9** outcompetes the intrinsic inhibitory effect of the unhindered azinyl nitrogen, affording *meta* borylated product **225** (Scheme 25 c). The Lewis basicity of the azinyl N can also be used for outer‐sphere direction in conjunction with a Lewis acid that is directly coupled to the bipyridine ligand, for example, **L13** (Scheme 25 e). Presumably, substrate coordination to these ligands outcompetes catalyst coordination, and this enables complementary C3 (*meta*) borylation of the parent pyridine scaffold at room temperature. Substituted pyridines are also viable substrates for this process. Interestingly, as shown by this example, a C‐2 substituent does not seem to block complexation, giving selective access to 2,5‐disubstituted pyridine products (Scheme 25 e).^[86]^

**Scheme 26:**
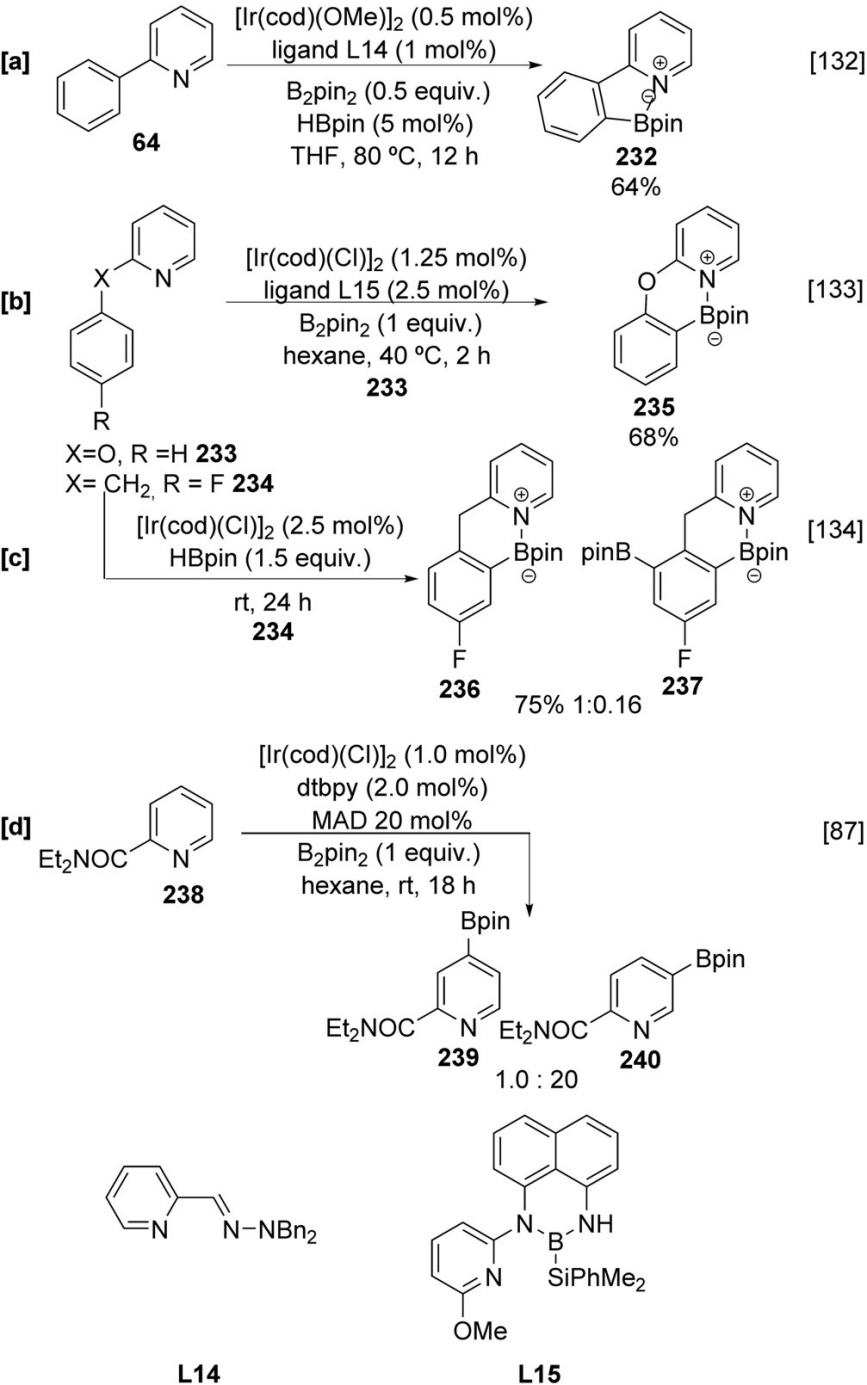
Inner‐sphere directed C−H borylation of 2‐substituted pyridines.

The azinyl nitrogen can also coordinate directly to the Ir metal centre in inner‐sphere systems enabling the selective borylation of pendant arene substituents (Scheme 26). Similar directed C−H activation processes of 2‐aryl pyridines are common with other metal catalysts, such as Ru.^[41]^ Using this approach, the electronic preference for borylation at the pyridine can be overcome in favour of reaction at the carbocyclic moiety.[^106^, ^132^] For example, hemilabile ligand **L14** efficiently facilitates borylation at the *ortho* position of the phenyl ring in 2‐phenylpyridine **64**, producing N‐B ylide **232** (Scheme 26 a).^[132]^ Likewise 2‐phenoxypyridine (**233**) undergoes borylation in the carbocyclic ring mediated by B‐Si ligand **L15**, (Scheme 26 b).^[133]^ In a related approach, 2‐benzyl pyridines act as a substrate, ligand, and inner‐sphere director and are selectively borylated in the carbocycle ring. Notably, fluorinated arene **234** selectively affords **236,** overcoming both the intrinsic reactivity of the pyridyl ring and the activating effect of a fluorine substituent toward *ortho* C−H activation. (Scheme 26 c).[^64^, ^134^] Nakao has exploited the coordinating ability of the azinyl nitrogen to direct the borylation to C‐4 using bulky aluminium Lewis acids which hinder access to the *meta* position. Surprisingly this still functions well in the presence of C‐2 substituents which appear not to hinder the crucial substrate Lewis acid binding (Scheme 26 d).^[87]^

### 4.4 Six Membered, Polycyclic, One Heteroatom (Quinolines and Isoquinolines)

**Scheme 27:**
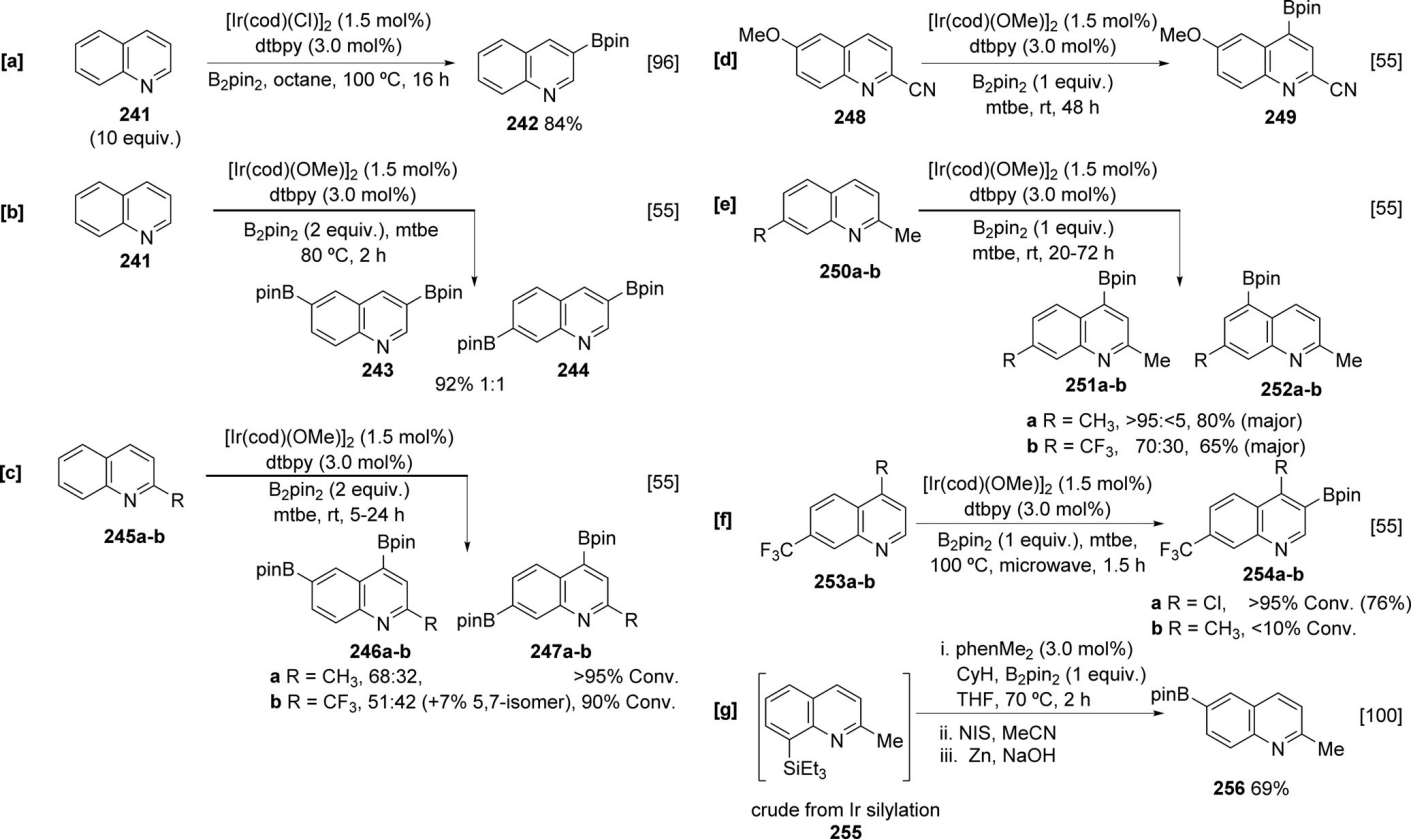
Ir‐catalysed C−H borylation of quinolines. NIS=*N*‐iodosuccinimide.

Although an azinyl heterocycle, unsubstituted quinoline **241** is an active substrate in the C−H borylation because the *peri* C‐8 C−H bond of the carbocyclic ring blocks inhibitory ligation to the active catalyst. As with other benzofused heteroarenes, quinoline is preferentially activated in the heteroaromatic ring. In the presence of excess heteroarene, selective monosubstitution at C‐3 can be obtained (Scheme 27 a), reflecting a combination of steric inhibition by the *peri* hydrogen at C‐5 and the inhibitory effect of the azinyl lone pair on activation at C‐2.^[96]^ In the presence of excess boron reagent, quinoline undergoes bisborylation at C‐3 and C6/C7 in a 1:1 ratio (Scheme 27 b).^[55]^ Unlike benzofused azoles, which show selectivity for reaction at C‐7, the analogous C‐8 position in quinoline is normally unreactive, providing further evidence for the repressive effect of a proximal azinyl N lone pair on C−H activation. As with other (hetero)arenes, the introduction of substituents leads to sterically controlled regioselectivity. For simple 2‐substituted quinolines the unhindered nature of the carbocyclic rings means that polyborylation is facile and the nature of the C‐2 substituent can affect the regiochemical outcome, with an increasing electron‐withdrawing ability leading to an increased degree of C‐7 substitution (Scheme 27 c).^[55]^ Substitution in the carbocyclic ring leads to a greater degree of control and further reveals the underlying role of electronic effects in these reactions. For example, whilst 2,6‐disubstituted quinolines are borylated exclusively at C‐4 (Scheme 27 d), which can be attributed to simple steric direction, 2,7‐disubstituted quinolines show varying selectivity, with more electron‐withdrawing groups leading to increased amounts of the C‐5 boronate ester **252 b** (Scheme 27 e). Since these positions are sterically equivalent this must reflect an electronic influence, and this is likely caused by the enhanced C−H acidity at C‐5 of the carbocyclic ring in the CF_3_‐containing substrate, **250 b**. Indeed, the calculated C−H acidities at C‐4 (38.6) and C‐5 (39.7) in **250 b** provide qualitative correlation with experimental site‐selectivity. As with other arenes, congested quinolines are viable substrates but require more forcing conditions, although the preference for reaction in the heteroaromatic ring remains. For example, all C−H sites in 4,7‐disubstituted quinolines are encumbered, so borylation is completely selective for C‐3 but the reaction of **253 a** bearing a chloro group at C‐4 is significantly more efficient than for the C‐4 methyl analogue (Scheme 27 f). As noted previously (see Section 4.2.1) a simple approach to deliver selective borylation in heteroarenes is to undertake sequential silylation, borylation and desilylation. Subjecting 2‐methyl quinoline to this process affords exclusively the 6‐borylated isomer **256** via silylated arene **255**. Since the corresponding 2,8‐dimethyl quinoline affords a complex mixture of mono‐ and diborylated products under the same conditions, this seems to reflect a combination of electronically and sterically driven selectivity. The silyl group may be selectively removed to afford a formal, selective, and otherwise difficult to achieve C‐6 borylation process (Scheme 27 g).^[100]^

**Scheme 28:**
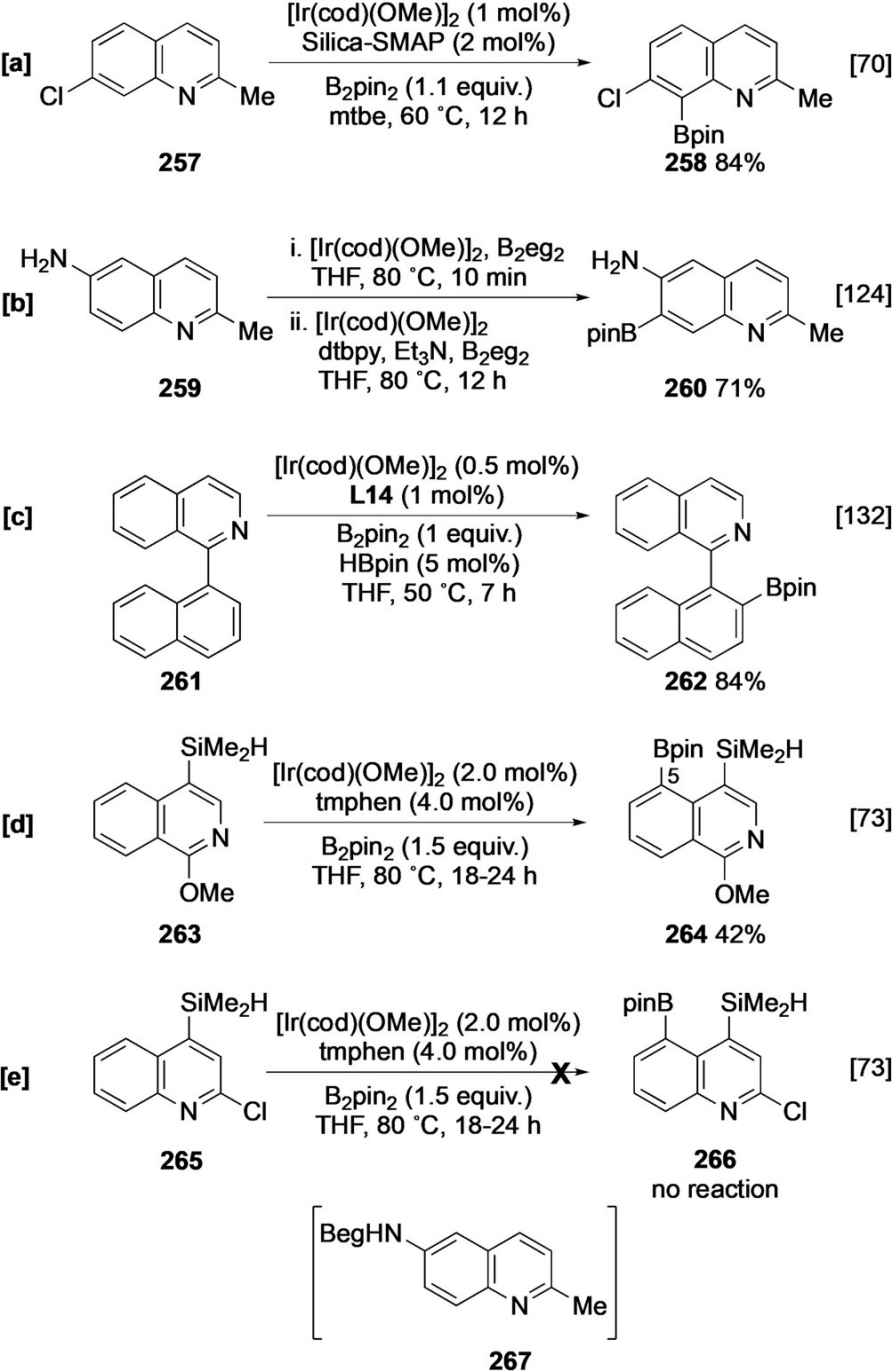
Directed borylation of quinolines.

By replacing dtbpy with the silica‐immobilised monodentate phosphine ligand Silica‐SMAP, Sawamura and co‐workers have elegantly exploited inner‐sphere coordination to selectively activate the C‐8 position in a range of 2‐substituted quinolines (Scheme 28 a).^[70]^ Silica‐SMAP directs borylation to the otherwise unreactive C‐8 position in a library of mono‐, di‐, and trisubstituted quinolines. Remarkably, the system affords C‐8 regioselectivity even in congested substrates, such as **257**, with a substituent at C‐7. Other groups can be used to direct regioselectivity; for example, aminoquinoline **259** has been shown to undergo *ortho‐*selective borylation at C‐7 via NHBeg intermediate **267** (Scheme 28 b).^[124]^ To date, the various other ingenious ligand‐controlled borylations have not been applied to quinoline and this may reflect the challenge of preparing suitably functionalised substrates.

Isoquinoline C−H borylation remains relatively unexplored, with the intrinsic regioselectivity undefined. This is probably attributable to catalyst inhibition by the heteroarene unless substituted at C‐1 and/or C‐3. However, some examples of directed borylation have been described. In a similar fashion to 2‐arylpyridines, hemilabile ligands facilitate selective remote borylation of 1‐aryl isoquinolines (Scheme 28 c),^[132]^ whilst hydrosilyl relay direction enables *peri* borylation of suitable 1‐substituted isoquinolines (Scheme 28 d).^[73]^ Curiously, similar chemistry using 2‐chloro‐4‐hydrosilylquinoline **265** was not viable (Scheme 28 e) and this result highlights the degree of substrate specificity that exist in these more complex systems that have multiple influences on reaction outcomes.

### 4.5 Five‐Membered, Monocyclic, Two Heteroatoms (Imidazoles, Pyrazole, Oxazole)

**Scheme 29:**
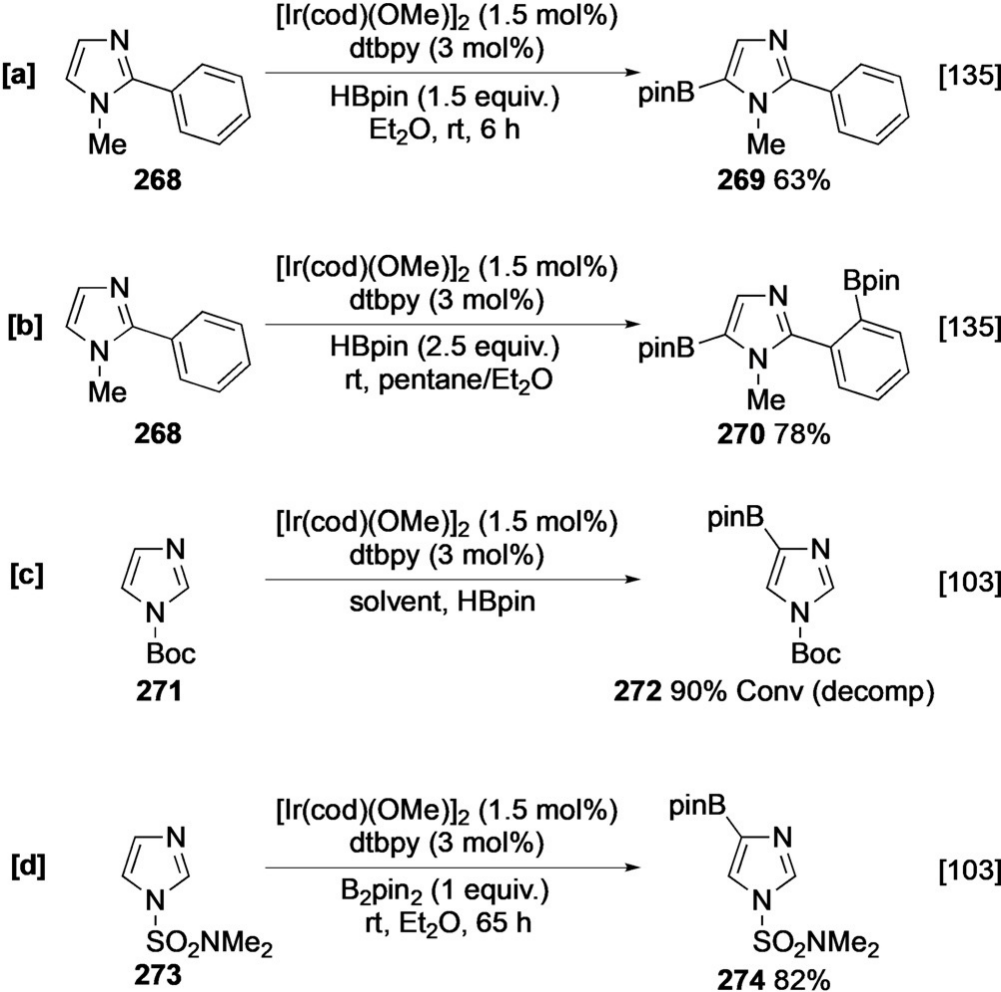
Borylation of imidazole derivatives.

Mirroring the effects observed with both pyrrole and pyridine, subject to steric accessibility, the borylation of imidazole, pyrazole, and oxazole generally occurs α to the oxygen atom or azole nitrogen and remote from the azinyl nitrogen atom. In general, the higher reactivity of these heterocycles together with the less congested relationship between substituents in five‐membered rings mean that steric effects are less pronounced, enabling positions with *ortho* substituents and even moderately sized doubly *ortho* substituted sites to be borylated (vide infra). The parent unprotected imidazole is not borylated, which is perhaps due to rapid N‐borylation to form the corresponding N‐Bpin adduct, leaving all C−H sites either sterically hindered or inhibited by the azinyl nitrogen. However, N‐methylated imidazoles undergo borylation efficiently at C‐5, and this is exemplified by the borylation of **268** in the presence of 1.5 equivalents of HBpin (Scheme 29 a). Interestingly, using 2.5 equivalents of HBpin, **268** undergoes a second borylation event at the *ortho* C−H site on the phenyl ring (Scheme 29 b), likely mediated by an outer‐sphere directing effect involving the azinyl N atom.^[135]^

As with 2‐subsituted pyridines, α‐azinyl borylation is normally disfavoured, although a combination of steric bulk and reduction of the azinyl electron density can allow this to occur. For example, blocking the azole N with either a Boc carbamate or dimethylsulfamoyl group enables α‐azinyl boronates **272** and **274** to be generated (Scheme 29 c,d).^[103]^ Whilst the Boc‐protected boronate was unstable, the higher electron‐withdrawing capacity of a sulfamoyl group rendered boronate **274** isolable.

**Scheme 30:**
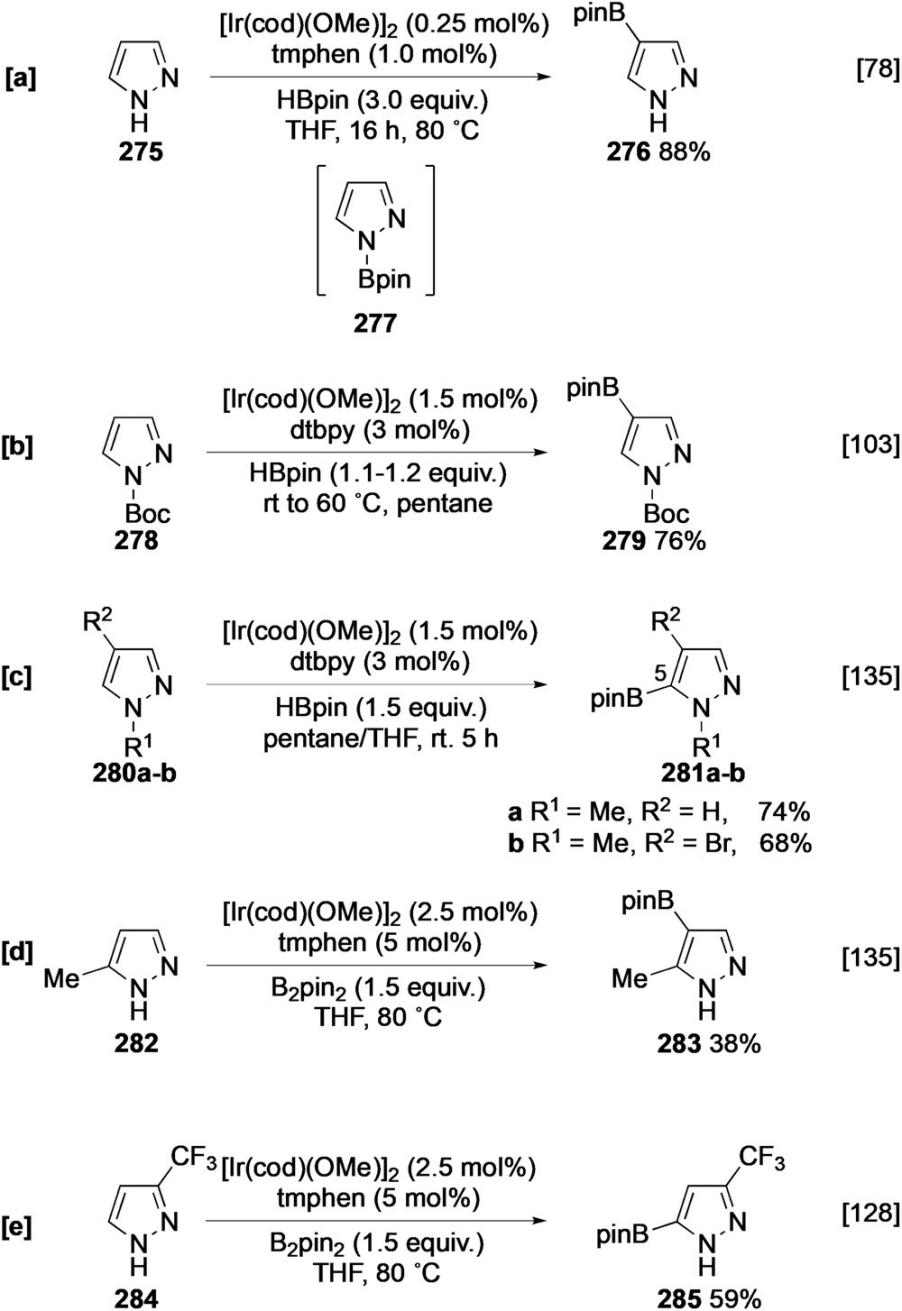
Borylation of pyrazole and its derivatives.

**Scheme 31:**
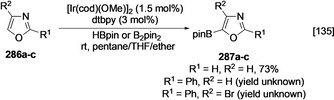
Borylation of oxazole derivatives.

In contrast to imidazole, unprotected pyrazole undergoes borylation at the β position owing to the rapid formation of bulky N‐Bpin species **277**, which sterically blocks α borylation (Scheme 30 a).^[78]^ Unlike indole, the lower p*K*_a_ of the pyrazole NH means that this does not require exogenous base as illustrated by the fact that **275** undergoes N‐borylation without catalysis. Other large nitrogen protecting groups such as Boc similarly direct borylation to the β position (Scheme 30 b), and recent results suggest that the β selectivity observed with **278** has an element of electronic control as the smaller methyl carbamate also leads to reaction at this position.[^103^, ^136^] Other small azole nitrogen substituents, for example, *N*‐methyl pyrazole (**280 a**) are selectively borylated at the α position reflecting the higher intrinsic reactivity of this C−H bond (Scheme 30 c). This selectivity is sustained even in the presence of an *ortho* bromine substituent (**280 b**). C‐5 substituted pyrazoles such as **282** also undergo N‐borylation and C‐borylation is selective for C‐4 (Scheme 30 d).^[135]^ However, tautomerisation in pyrazoles is strongly modulated by the substituents and, in contrast to free N−H imidazoles, 3‐trifluoromethylpyrazole **284** is borylated at C‐5 and not at C‐4, with steric and electronic selectivity working in parallel (Scheme 30 e).^[128]^ Significantly fewer oxazole derivatives have been reported as substrates for C−H borylation, although some examples are shown in Scheme 31. In these substrates, there is a normal preference for borylation at the most acidic site α to the O atom.^[135]^ Somewhat surprisingly, directed borylation, inner‐ or outer‐sphere, of imidazole, pyrazole, or oxazole derivatives has yet to be described.

### 4.6 Five‐Membered Polycyclic Heterocycles with Two Heteroatoms

#### 4.6.1 Indazole

**Scheme 32:**
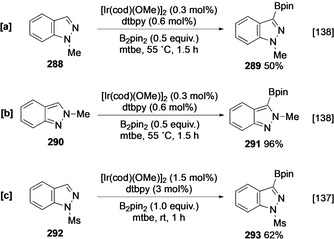
C−H borylation of indazoles. Ms=methanesulfonyl.

**Scheme 33:**
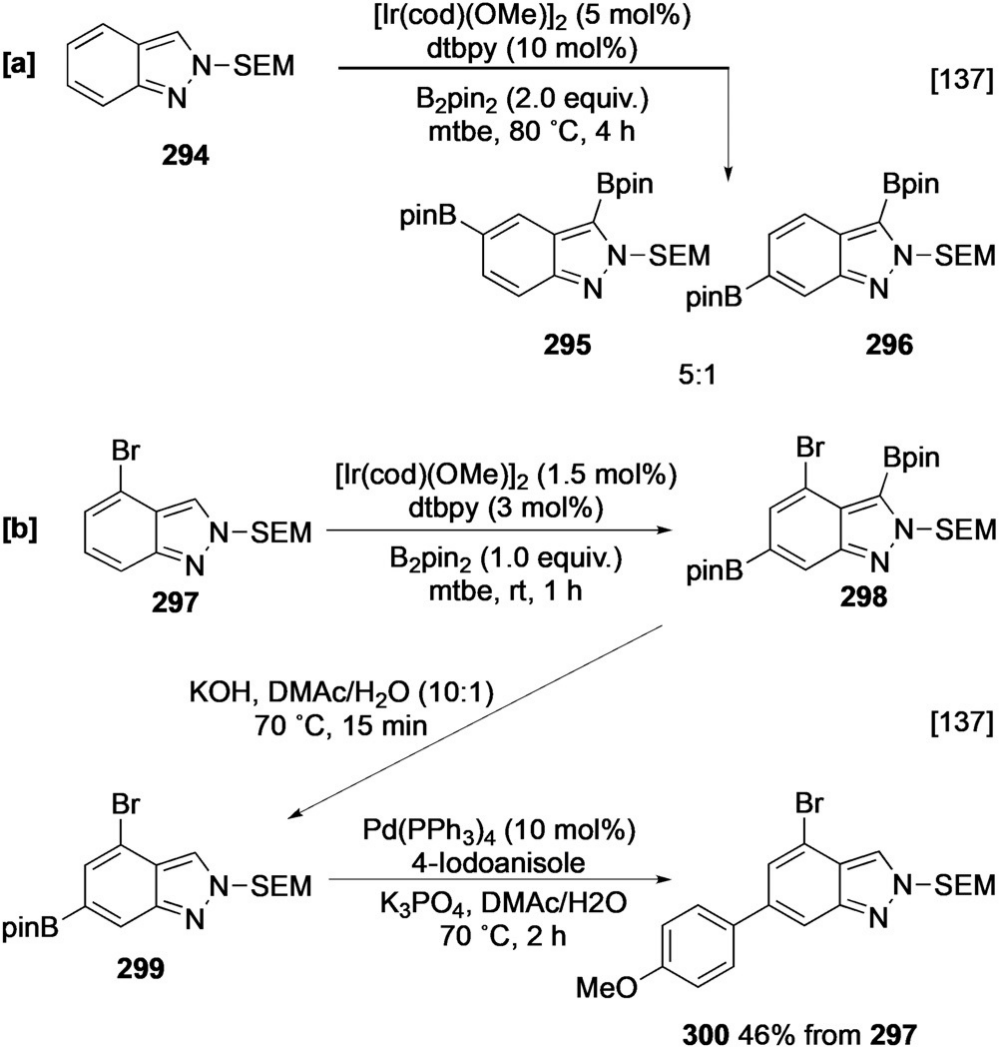
Multidirectional functionalisations of 2*H*‐indazoles. SEM=2‐(trimethylsilyl)ethoxymethyl.

As with imidazole, the borylation of unprotected NH indazole is not effective. This is surprising, as the structurally similar heteroarene pyrazole is an active substate and is borylated at C‐4 following initial N−H borylation. The lack of indazole borylation has been suggested to be due to the inhibitory effect of the azinyl N on the catalyst.[^137^, ^138^] Alternatively, the parallels with imidazole might also suggest it is either an inability to N‐borylate or, more likely, an instability of the N‐borylated species that is critical. In support of this latter idea, the more stable N‐substituted indazole derivatives undergo facile borylation. Significantly, both *N*‐methyl‐1*H*‐indazole (**288**) and *N*‐methyl‐2*H*‐indazole (**290**) show complete selectivity for the heteroaromatic moiety (Scheme 32 a,b). Moving from N‐Me to larger substituents, such N‐Boc, N‐tetrahydropyranyl (N‐THP), N‐2‐(trimethylsilyl)ethoxymethyl (N‐SEM) or N‐3,5‐dimethylbenzyl does not alter this regioselectivity for either 1*H*‐ or 2*H*‐indazoles (Scheme 33). This provides further evidence for a difference in five‐ and six‐membered heterocycles, with borylation occurring adjacent to both the *peri* C‐4 substituent and azinyl nitrogen (Scheme 33 b). This reflects both the lower steric demand of *ortho* substituents and a lower inhibitory effect of the azinyl nitrogen which is strongly influenced by the electron‐withdrawing effect of the azole nitrogen (p*K*_a_(indazolium)=1.25).

The azinyl nitrogen effect is not completely ablated, as borylation of the 2*H*‐indazole isomers, which avoid this inhibitory interaction, is much more rapid and the corresponding C‐3 boronates undergo protodeborylation more slowly. The latter issue can be ameliorated through the use of the more electron‐withdrawing N‐Ms group, as in **292**, which stabilises boronate **293**, permitting isolation following silica gel column chromatography in 62 % yield (Scheme 32 c). As with pyridines, the introduction of blocking groups (e.g. halogens) enables more complex sequences involving polyborylation and selective reaction, for example, cross‐coupling and deborylation, giving access to other regioisomers. For example, at elevated temperature in the presence of 2 equivalents of B_2_pin_2_, *N*‐SEM‐2*H*‐indazole (**294**) iteratively undergoes bisborylation, initially at C3 and then at C‐5/C‐6, to afford a 5:1 mixture of products (Scheme 33 a). Substitution at C‐4, as in **297** leads to the second C−H activation occurring exclusively at C‐6 (Scheme 33 b). In this case, exploiting the destabilising effect of the azinyl nitrogen enables selective protodeborylation at C‐3 leading to a formal C‐6 monoborylation affording **299**.

#### 4.6.2 Benzoxazole, Benzothiazole, and Benzimidazole

**Scheme 34:**
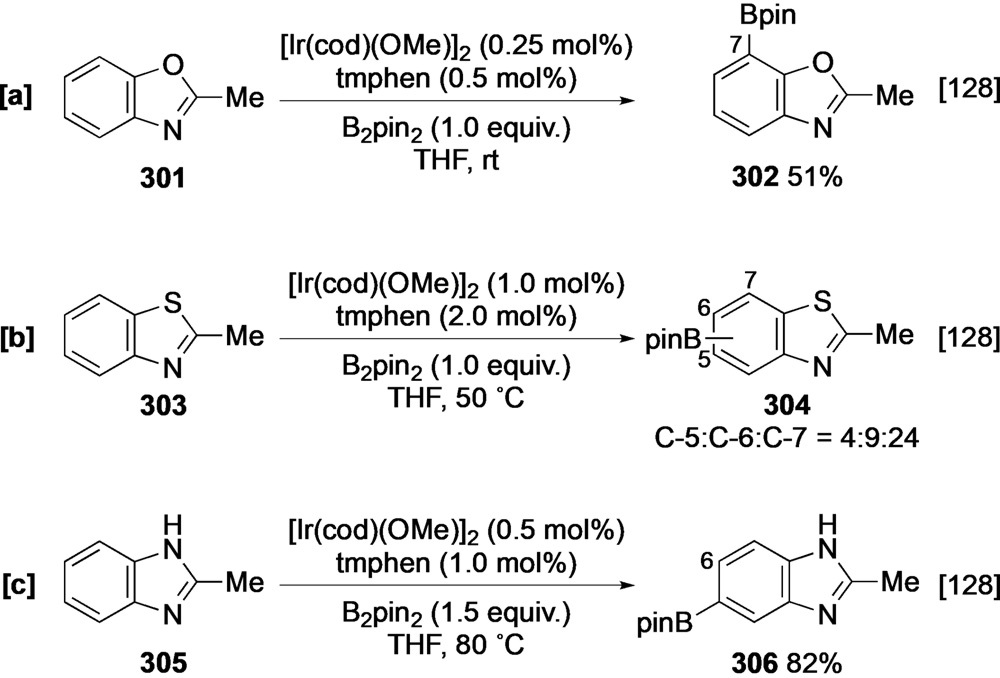
C−H borylation of benzoxazole, benzothiazole, and benzimidazole derivatives.

The borylation of benzoxazoles, benzothiazoles, and benzimidazoles requires a substituent at C‐2, potentially to prevent substrate ligation to the catalyst through the azinyl nitrogen. The reasons for this are not immediately obvious, as oxazole itself is an active substrate (see Section 4.5), although this may further reflect the higher reactivity of heterocyclic C−H bonds when compared with those in carbocyclic rings. In 2‐methylbenzoxazole (**301**), borylation selectively occurs at C‐7 *ortho* to the O atom (Scheme 34 a), and other derivatives with substituents at C‐4 or C‐5 also show this regioselectivity.^[128]^ This is consistent with the *ortho* selectivity observed in benzodioxole **36** (see Section 3.1), and correlates to the enhanced C−H acidity of this site. Mirroring the lower electronegativity of sulfur, 2‐methylbenzothiazole (**303**) is comparably less active towards C−H borylation and displays poorer selectivity (Scheme 34 b). The requirement for elevated temperature and catalyst loadings likely reflects the catalyst‐deactivating effect of sulfur, and parallels can be drawn with thiophenes, which are less active than pyrrole and furan for this reason (see Section 4.1.1). In contrast to both these heterocycles, 2‐methylbenzimidazole (**305**) efficiently undergoes distal borylation at C‐5 (Scheme 34 c). The lack of *ortho* (C‐4/C‐7) reactivity can be attributed to an azinyl N effect in analogy to the inhibition of C‐8 activation in quinoline, although the contribution of an N‐Bpin adduct, which also sterically hinders the *peri* position, cannot be discounted. The latter possibility is supported by the requirement for increased equivalents of B_2_pin_2_ for efficient borylation of benzimidazoles.

### 4.7 Six‐Membered Heterocycles, Two Heteroatoms

#### 4.7.1 Diazines

**Scheme 35:**
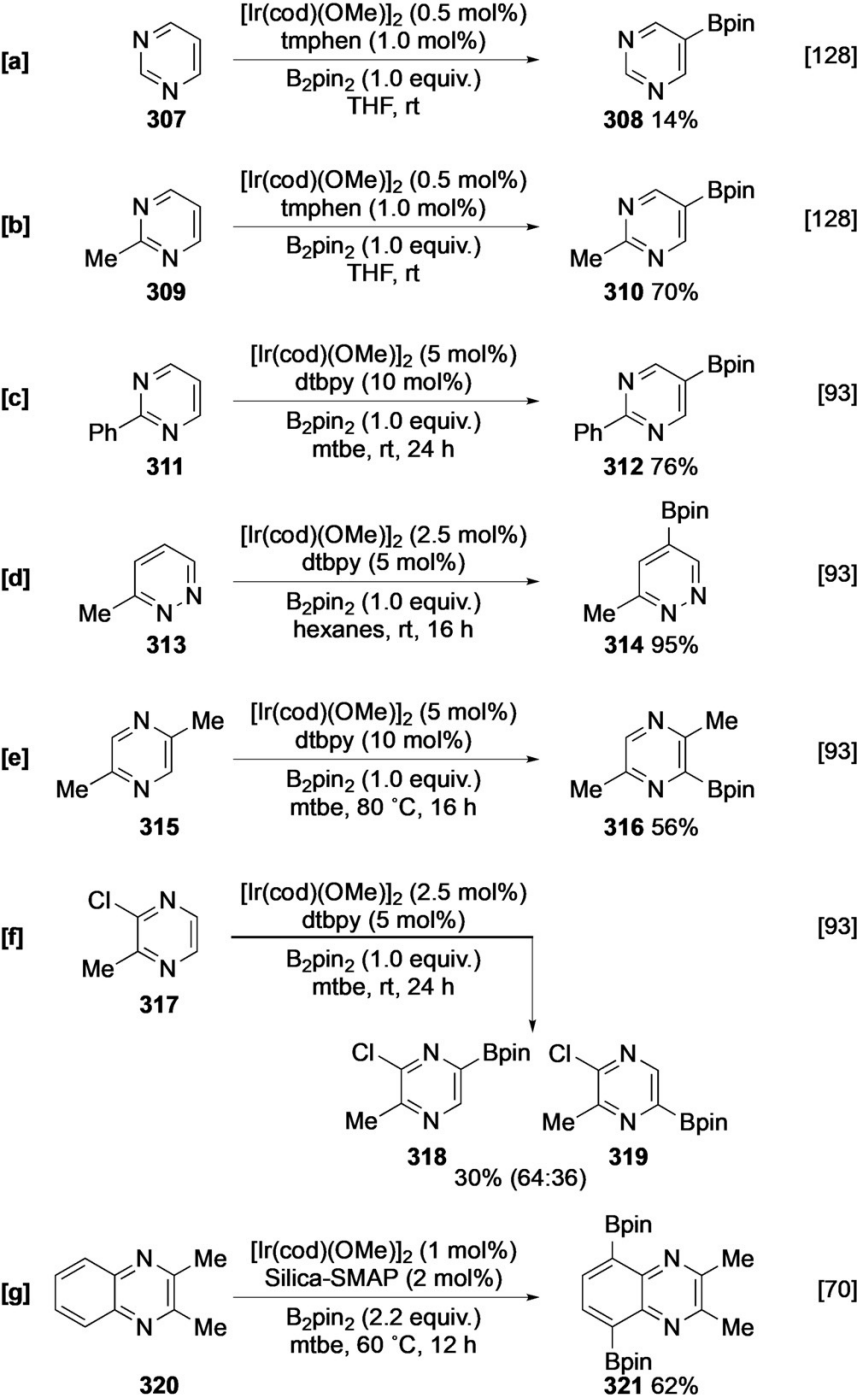
C−H borylation of diazines.

The presence of two N ring atoms renders diazines less basic than pyridine. This, coupled with the enhanced C−H acidity, affords greater intrinsic activity. Indeed, parent pyrimidine **307** is borylated at room temperature using tmphen as a ligand, albeit in low (NMR) conversion.^[128]^ In analogy to the C−H borylation of other azinyl systems, the catalyst avoids the α‐azinyl positions and delivers C‐5 functionalised boronate **308** selectively (Scheme 35 a). Blocking ligation of diazine substrates with substituents improves reactivity, and this may be seen in the comparably more efficient borylation of 2‐substituted pyrimidines (Scheme 35 b,c). As with other heterocycles, and exemplified by the reaction of 2‐phenylpyrimidine (**311**; Scheme 35 c), activation of C−H bonds within the heterocyclic ring occurs more rapidly than in carbocyclic arenes. Likewise, the borylation of 6‐methylpyridazine **313** is efficient and selective for C‐4 (Scheme 35 d). In contrast to pyridines, substituted pyridazines have been shown to undergo borylation at the α‐azinyl C−H bond without necessarily requiring electron‐withdrawing substituents. For example, pyridazine **315** is efficiently borylated at C‐6, despite the presence of both an *ortho* azinyl N and an *ortho* methyl group (Scheme 35 e). Interestingly, the 2‐chloro‐3‐methylpyridazine (**317**) is preferentially borylated *meta* to the chlorine atom, as this nitrogen carries lower electron density (Scheme 35 f).^[93]^ Finally, in parallel with the transformation of quinolines, the borylation of quinoxaline has been reported with Silica‐SMAP, affording bisborylated product **321** via inner‐sphere coordination of the azinyl N atoms (Scheme 35 g).^[70]^

#### 4.7.2 Azaborine and Borazaronaphthalene

**Scheme 36:**
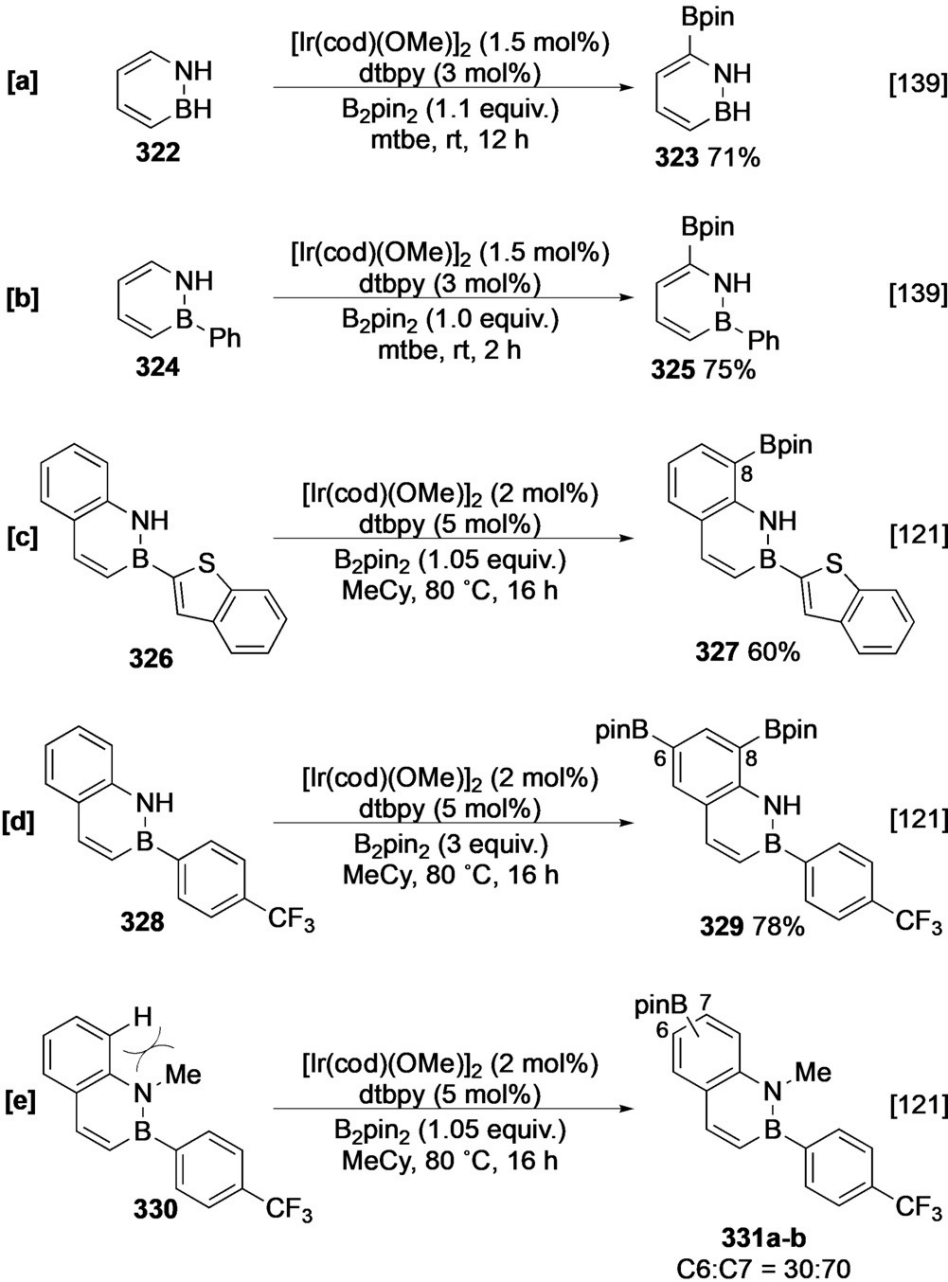
C−H borylation of boron‐containing heteroarenes.

Boron–nitrogen heterocycles such as borazine and 2,1‐borazaronaphthalene are isostructural with classical arenes and can undergo Ir‐catalysed C−H borylation. In analogy to other azoles, borazine **322** undergoes borylation selectively α to N at C‐6 (Scheme 36 a).^[139]^ Under these conditions, an aryl substituent on the boron atom is not affected and this selectivity correlates well with calculated gas‐phase acidity (Scheme 36 b). 2,1‐Borazaronaphthalenes are benzofused analogues of borazine, and this motif differs from other benzofused heteroarenes in that C−H borylation exhibits selectivity for the carbocyclic ring. For instance, the carbocycle of **326** is more reactive than both the azaborine and benzothiophene rings and undergoes selective borylation at C‐8 (Scheme 36 c).^[121]^ In parallel to the chemistry observed with indole,^[116]^ it is possible that the N−H group plays a role in enabling directed borylation through an inner‐sphere effect. However, calculations have shown that this site has the greatest anionic charge stabilisation, suggesting that the selectivity may be intrinsic in origin. Moreover, the notion that, in borazaronaphthalenes, the carbocyclic ring is more reactive is reinforced by the fact that both bisborylation of **328** and borylation of the N‐methylated analogue **330** occurs in the carbocyclic and not the heterocyclic ring, albeit at what are the most accessible C−H bonds (Scheme 36 d,e).

### 4.8 Fused Heterocyclic Rings with Multiple Heteroatoms

Borylation of fused heterocycles containing multiple heteroatoms is possible, although fewer examples have been reported. In general, a similar profile of reactivity can be established in which selectivity is a balance of accessibility and intrinsic C−H activity (C−H acidity/C−Ir bond strength) countered by the inhibitory effect of a proximal and or Lewis basic azinyl nitrogen.

#### 4.8.1 Pyrazolo‐, Imidazo‐, and Tetrazolopyridine

**Scheme 37:**
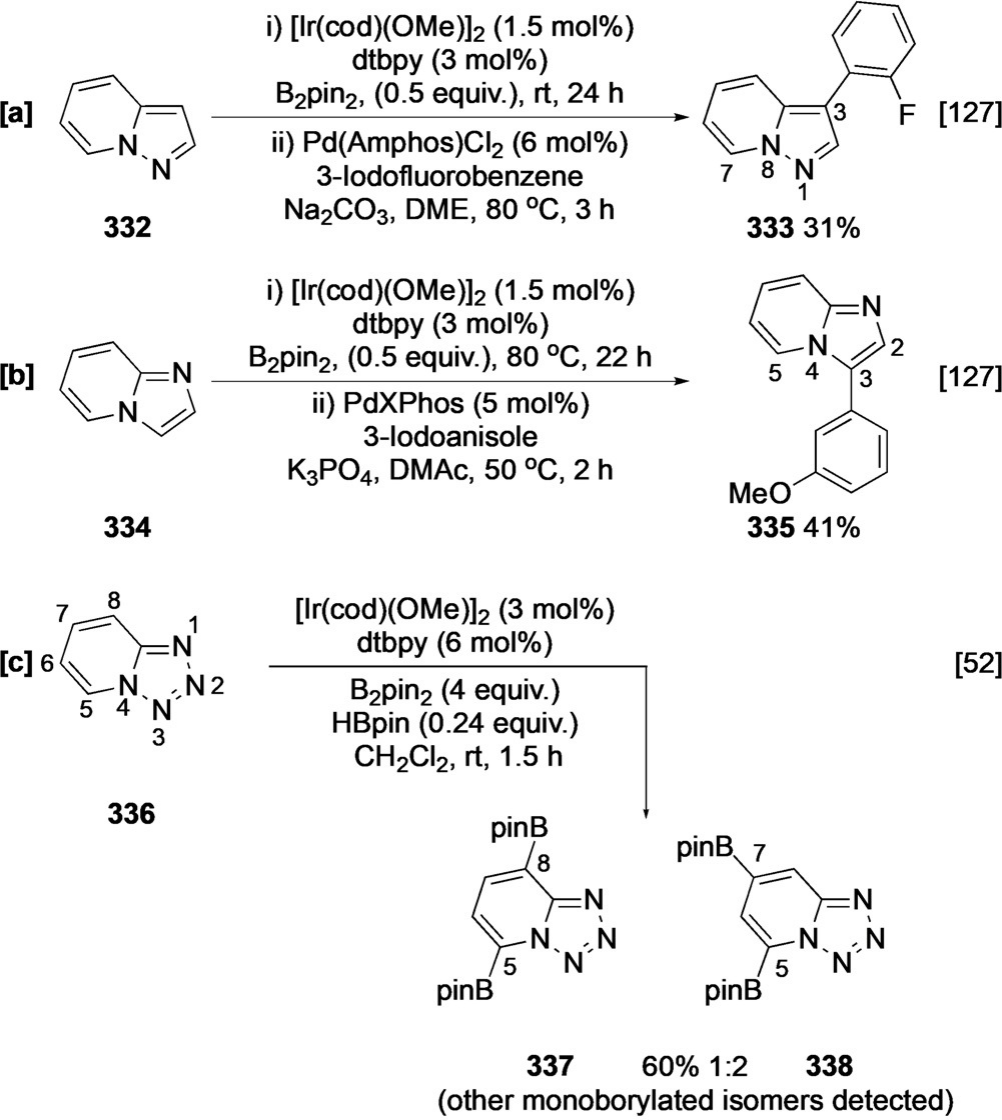
C−H borylation of imidazo‐, pyrazolo‐ and tetrazolopyridine. Amphos=bis(di‐*tert*‐butyl(4‐dimethylaminophenyl)phosphine, DME=dimethyl ether, XPhos=2‐dicyclohexylphosphino‐2′,4′,6′‐triisopropylbiphenyl.

Pyrazolo[1,5‐*a*]pyridine (**332**) can be envisaged as a 1,5‐disubstituted pyrazole, with C−H borylation exhibiting C‐5 selectivity, avoiding the α azinyl position (Scheme 37 a). Similarly, imidazo[1,2‐*a*]pyridine (**334**) can be envisaged as a 1,2‐disubstituted imidazole, with C−H borylation exhibiting C‐3 selectivity α to the azole‐like N‐4 nitrogen and avoiding the C‐2 α azinyl position (Scheme 37 b). No C−H borylation is observed in the six‐membered ring, and following tandem Suzuki–Miyaura cross‐coupling, both substrates display C−H borylation selectivities that mirror their respective five‐membered heteroaromatic analogues.^[127]^ The tetrazolopyridine **336** can only be borylated in the six‐membered ring. Mirroring the regioselectivity of other azoles, C‐5 functionalisation α to the azole‐like N‐4 nitrogen is most favoured. With excess boron reagent, C‐7 functionalisation occurs more rapidly than at C‐8, presumably reflecting a balance between steric accessibility, inhibition by the N‐1 azinyl lone pair, and activation by the *para* C‐5 Bpin group. Interestingly, the borylation of **336** was far more efficient in CH_2_Cl_2_ than in THF or MTBE despite the fact that chlorinated solvents are seldom employed in iridium C−H borylation as they are generally inferior to alkanes and ethers.

#### 4.8.2 Azaindole, Azaindazole, and Deazapurine

**Scheme 38:**
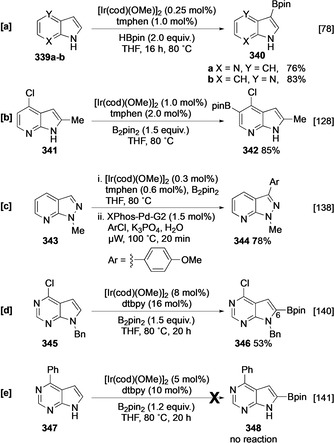
C−H borylation of fused heterocycles. μW=microwaves.

In free unprotected azaindoles **339 a**,**b**, N−H acidity is elevated by the presence of the azine N, facilitating spontaneous N−H borylation with HBpin. Consequently, the corresponding N‐Bpin adduct blocks the C‐2 position, leading to C‐3 selective borylation (Scheme 38 a).^[78]^ In general, the five‐membered ring is intrinsically more reactive and is the site of C−H activation unless steric hindrance is introduced (Scheme 38 b).^[128]^ The C−H borylation of *N*‐methylazaindazole (**343**) is similarly C‐3 selective and affords **344**, reflecting the enhanced C−H acidity of the pyrazole ring hydrogens (Scheme 38 c).^[138]^ The presence and nature of the substituents and boron source play a role in determining activity; for example, the borylation of N‐benzylated 7*H*‐pyrrolo[2,3‐*d*]pyrimidine (*N*‐benzyldeazapurine) **345** is selective for the heteroarene (Scheme 38 d), whereas borylation of C‐4 substituted free N−H deazapurine **347** using B_2_pin_2_ does not afford any C−H borylation products (Scheme 38 e).[^140^, ^141^]

## 5 Summary

Organoboron compounds are versatile intermediates and the selective generation of these is paramount for many applications. Almost twenty years after its inception, Ir‐catalysed aromatic C−H borylation remains the state‐of‐the‐art methodology for the regioselective installation of arene C−B bonds. In general, the crowded nature of the catalyst permits sterically controlled borylation of carbocyclic C−H bonds, and the least hindered sites are generally the most reactive. Electronic effects, although contributing to the reaction outcome, are a relatively minor component and, generally, only observed at lower temperatures. In contrast, although the site‐selectivity observed in the borylation of heteroarenes carries a degree of steric control, a significant contribution from electronic effects is apparent, as evidenced by the observation that more congested C−H bonds can undergo selective borylation. Factors such as Ir−C bond strength, relative anionic stabilisation, and C−H acidity, in conjunction with sterics, all contribute more overtly to the outcome of this transformation and should be considered in order to predict and understand heterocycle borylation selectivities. Moreover, whilst the intrinsic steric‐regulated selectivities of carbocyclic aromatic C−H borylation can be altered using ligand‐based directing effects, the multiple factors observed in many heterocyclic systems complicate the application of these strategies to such substrates.

In conclusion, the increasing number of reports describing the application of Ir C−H borylation to new heterocyclic systems demonstrate the importance of this methodology. Selectivity remains a challenging aspect and we hope that this review shall serve as a useful resource for predicting the intrinsic borylation regioselectivity of these and related heterocyclic systems. Looking forward, much of this chemistry has been achieved using a relatively limited set of ligands and boron reagents, and the development of new systems that enable greater scope and control in these transformations remains an important synthetic objective.^[92]^

## Conflict of interest

The authors declare no conflict of interest.

## Biographical Information

*Jay Wright earned an MSc in Chemistry from the University of Birmingham (UK) in 2015. During his final year, he studied the synthesis of N‐heterocyclic carbene ligands under the supervision of Dr. Paul Davies. He then moved to the University of Durham (UK) to conduct his doctoral studies on Ir‐catalysed C−H borylation of heteroarenes under the supervision of Prof. Patrick G. Steel, graduating in 2020. Currently, he is a postdoctoral research fellow at the University of Michigan, (USA), developing new transition metal promoted radiofluorination methods under the supervision of Prof. Peter J. H. Scott*.

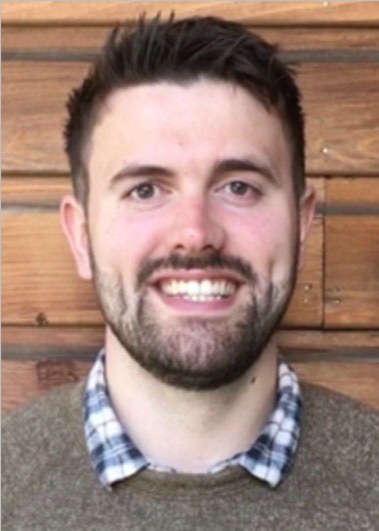

## Biographical Information

*Peter Scott obtained his BSc from Loughborough University (UK) and his PhD, under the mentorship of Prof. Patrick Steel, from Durham University. He undertook postdoctoral research at SUNY Buffalo under Prof. Huw Davies, and the University of Michigan with Prof. Michael Kilbourn. He joined the faculty at Michigan in 2009 and is currently an Associate Professor of Radiology and Director of the PET Centre. He is a Fellow of the Royal Society of Chemistry and received a Distinguished Investigator Award from the Academy for Radiology & Biomedical Imaging Research in 2019*.

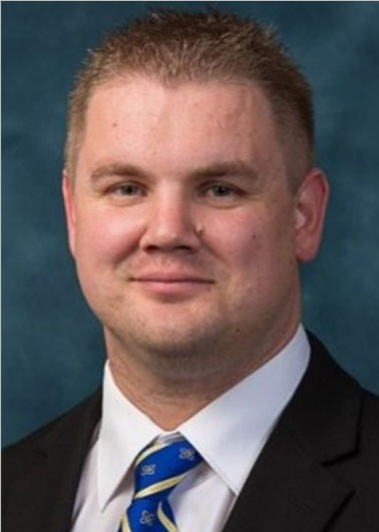

## Biographical Information

*Patrick Steel undertook his undergraduate and post‐graduate training with Prof. Jim Thomas at the University of Oxford (UK). Following a NATO‐SERC postdoctoral fellowship with Prof Gilbert Stork at Columbia University (USA), he joined the staff at Durham University where he is currently a professor of organic chemistry and chemical biology. He is interested in problems in organic synthesis and chemical biology with particular focus in neglected tropical diseases and the chemical biology of plants. He was Head of the Organic Chemistry Section at Durham University 2007–2013 and was an elected council member of the Royal Society of Chemistry, Chemistry Biology Interface Division 2013–2019*.